# Axonal transport during injury on a theoretical axon

**DOI:** 10.3389/fncel.2023.1215945

**Published:** 2023-08-11

**Authors:** Soumyadeep Chandra, Rounak Chatterjee, Zachary T. Olmsted, Amitava Mukherjee, Janet L. Paluh

**Affiliations:** ^1^Electrical and Computer Science Engineering, Purdue University, West Lafayette, IN, United States; ^2^Department of Electronics, Electrical and Systems Engineering, University of Birmingham, Birmingham, United Kingdom; ^3^Nanobioscience, College of Nanoscale Science and Engineering, State University of New York Polytechnic Institute, Albany, NY, United States; ^4^Department of Neurosurgery, Ronald Reagan UCLA Medical Center, University of California, Los Angeles, Los Angeles, CA, United States; ^5^School of Computing, Amrita Vishwa Vidyapeetham (University), Kollam, Kerala, India

**Keywords:** traumatic brain injury, axonopathy, neurotransmission, microtubules, kinesins, TASEP-LK

## Abstract

Neurodevelopment, plasticity, and cognition are integral with functional directional transport in neuronal axons that occurs along a unique network of discontinuous polar microtubule (MT) bundles. Axonopathies are caused by brain trauma and genetic diseases that perturb or disrupt the axon MT infrastructure and, with it, the dynamic interplay of motor proteins and cargo essential for axonal maintenance and neuronal signaling. The inability to visualize and quantify normal and altered nanoscale spatio-temporal dynamic transport events prevents a full mechanistic understanding of injury, disease progression, and recovery. To address this gap, we generated DyNAMO, a Dynamic Nanoscale Axonal MT Organization model, which is a biologically realistic theoretical axon framework. We use DyNAMO to experimentally simulate multi-kinesin traffic response to focused or distributed tractable injury parameters, which are MT network perturbations affecting MT lengths and multi-MT staggering. We track kinesins with different motility and processivity, as well as their influx rates, in-transit dissociation and reassociation from inter-MT reservoirs, progression, and quantify and spatially represent motor output ratios. DyNAMO demonstrates, in detail, the complex interplay of mixed motor types, crowding, kinesin off/on dissociation and reassociation, and injury consequences of forced intermingling. Stalled forward progression with different injury states is seen as persistent dynamicity of kinesins transiting between MTs and inter-MT reservoirs. DyNAMO analysis provides novel insights and quantification of axonal injury scenarios, including local injury-affected ATP levels, as well as relates these to influences on signaling outputs, including patterns of gating, waves, and pattern switching. The DyNAMO model significantly expands the network of heuristic and mathematical analysis of neuronal functions relevant to axonopathies, diagnostics, and treatment strategies.

## 1. Introduction

Traumatic brain injury (TBI) and the associated long-term disease progression known as chronic traumatic encephalopathy (CTE) continue to be the leading causes of mortality and morbidity worldwide, with implications for Alzheimer's disease and Parkinson's disease (Daneshvar et al., 2015). Trauma pathophysiology includes damage to neurons such as shearing, crush damage, and stretch injuries that result in axonal pathologies at the earliest stages of the disease. Distortion of axonal cytoskeleton structure and varicosities, which are axonal protrusions similar to beads on a string, may also be evident along axon length (McKee et al., 2013). Axonal transport is required for neuron homeostasis, and neural signaling and disruptions in the process are increasingly associated with a variety of neurological disorders (Sleigh et al., 2019). Shared neuropathological hallmarks such as the transactivation response (TAR) DNA-binding protein 43 (TDP-43) are seen not only in TBI but also in other neurodegenerative pathologies, such as Amyotrophic lateral sclerosis (ALS), for which axonal transport defects are common (Baldwin et al., 2016). TDP-43 is a multifunctional RNA-binding protein with roles in mRNA transport in axons and dendrites and contributes to localized translation in pre-synaptic neurons for rapid signaling and neuronal homeostasis (Ling, 2018; Nagano et al., 2020). Regulation of TDP-43 is linked to axonal survival and neurodegeneration in visual circuits (Shigeoka et al., 2016) as well as additional roles in the structure and function of dendritic spines in Drosophila and mice (Lu and Vogel, 2009; Fogarty et al., 2016; Handley et al., 2016). While disease hallmarks such as TDP-43 highlight the outcomes of disrupted axonal transport, theoretical models such as DyNAMO can now provide a detailed mechanistic understanding of the nano- to microscale processes that escape detection due to the limitations of live cell imaging at this scale. The DyNAMO theoretical axon is the first computational platform designed to enable studies of altered dynamics of multiple motor proteins in axonal injury scenarios, including visualization, classification, and quantification of transiting events and outputs to drive long-term advancements in treatments for TBI and other axonopathies.

In the three decades since the discovery of neuronal kinesin and fast axonal transport (Smith, 1980; Tsukita and Ishikawa, 1980; Vale et al., 1985), progress toward nanoscale visualization of dynamics along axons remains challenging (Surana et al., 2020). Biological studies of fast axonal transport in living cells, which initially relied on techniques such as video-enhanced contrast-differential interference contrast microscopy (Allen et al., 1982; Song et al., 2016), have evolved to apply correlative live cell and super-resolution microscopy (Bálint et al., 2013), as well as an advanced and growing toolkit of technical approaches (Surana et al., 2020), but still remain challenging for interpreting and evaluating interactions of multiple types of biomolecules at nanoscale resolution. In contrast, detailed imaging of fixed samples has led to a dramatic new realization of the complex and layered biological infrastructure of the axon that includes three interacting cytoskeletons, containing discontinuous, staggered, polar MT bundles (Yamada et al., 1971), actin waves (Ruthel and Banker, 1999), actin rings (Xu et al., 2013; Vassilopoulos et al., 2019), actin hotspots and trails (Ganguly et al., 2015), and neurofilaments (Trojanowski et al., 1986; Nixon and Shea, 1992; reviewed by Leterrier et al., 2017; Papandréou and Leterrier, 2018; Hahn et al., 2019; Leterrier, 2021), as well as numerous interacting motor and non-motor binding proteins (Hirokawa and Takemura, 2005; Conde and Cáceres, 2009; Hirokawa et al., 2009) essential to axonal functions. Advanced mathematical models are critical for bridging experimental gaps, such as nanoscale dynamics, while incorporating static information from high-resolution images on fixed samples or obtained by super-resolution methods on living cells into the theoretical heuristic. Few computational models exist to address axonopathies. A model allowing simulations of the viscoelasticity of the MT-associated protein tau, MAPT, was developed relevant to Alzheimer's disease and axonal stretch injuries (Ahmadzadeh et al., 2014, 2015; Barbier et al., 2019). Banerjee et al. (2018) modeled multiple neuron compartmentalized functions as a nanoscale end-to-end communication system, enabling experimental data from multiple biological studies to be evaluated to compare amyloid-beta neurotoxicity at membranes, receptors, and internal calcium stores relevant to Alzheimer's disease. The DyNAMO theoretical axon is a significant step in providing a detailed framework that significantly expands the ability to interrogate a diverse set of parameters relevant to axonal MT injury, infrastructure perturbations, and impacts on motor-based axonal transport relevant to neurological disease (Sleigh et al., 2019).

To model axonal transport, including non-equilibrium statistical mechanics and diffusion behaviors of multiple motors on axonal MT bundles, DyNAMO applies a totally asymmetric simple exclusion process, TASEP (Spohn, 1991; Derrida, 1998; Derrida and Evans, 2009), and Langmuir kinetics (LK). TASEP is a cross-disciplinary framework that restricts events to a unidirectional driven diffuse system relevant to diverse biological applications of intracellular molecular transport as well as the fields of chemistry and physics. LK algorithms, when integrated with TASEP, allow the capture of diffusive absorption/desorption dynamics, such as reattachment/detachment of motor proteins from MTs and inter-lane transition (Wang et al., 2007) as well as more complex scenarios in DyNAMO. Generalized transport studies of single or dual MTs have revealed the potential of combined TASEP-LK analysis on high- and low-density particle trafficking (Parmeggiani et al., 2003, 2004; Nishinari et al., 2005; Wang et al., 2007). DyNAMO significantly builds on these approaches to incorporate a rich set of parameters for dense capture of information, quantification, and co-evaluation relevant to axonal transport. This includes staggered discontinuous MT bundles, dynamic events of multiple motors with motor-specific motility parameters, and motor-type MT crowding, dissociation, and reassociation dynamics at site-specific locations along the theoretical axon, never co-evaluated. DyNAMO provides multiple types of quantifiable readings resulting from MT injury for the motor types navigating the theoretical axon, including input, stalling, progression patterns, and outputs. The roles and impact of varying kinesin types and their motility parameters in *in vivo* axon transport (Brunner et al., 2004; Verbrugge et al., 2009; Lo et al., 2011; Sun et al., 2011; Jenkins et al., 2012; Lessard et al., 2019), including axonal distribution (Hirokawa et al., 2009), remain unknown. Advanced theoretical axon models, such as DyNAMO, will benefit the understanding of axonal mechanisms and aid the analysis of brain injury pathologies and axonopathies that impact axonal MT structure and neuronal signaling. DyNAMO provides the ability to simulate MT length and multi-MT staggering injury changes in axonal MT cytoskeleton architecture along with impacts on the progression of multiple kinesins of differing motility and processivity. DyNAMO expands our understanding of axonal injury events and provides a means to spatio-temporally characterize and quantify associated transport impacts. This information and the continued advancement of neuronal theoretical models are anticipated to have numerous benefits, including mechanistic and biomarker discovery to distinguish minor vs. severe changes in axonal transport and other mechanistic considerations in designing future diagnostics and therapeutics for axonopathies.

## 2. Materials and methods

### 2.1. Development of a framework to capture complex axonal parameters

**Figure 1:**
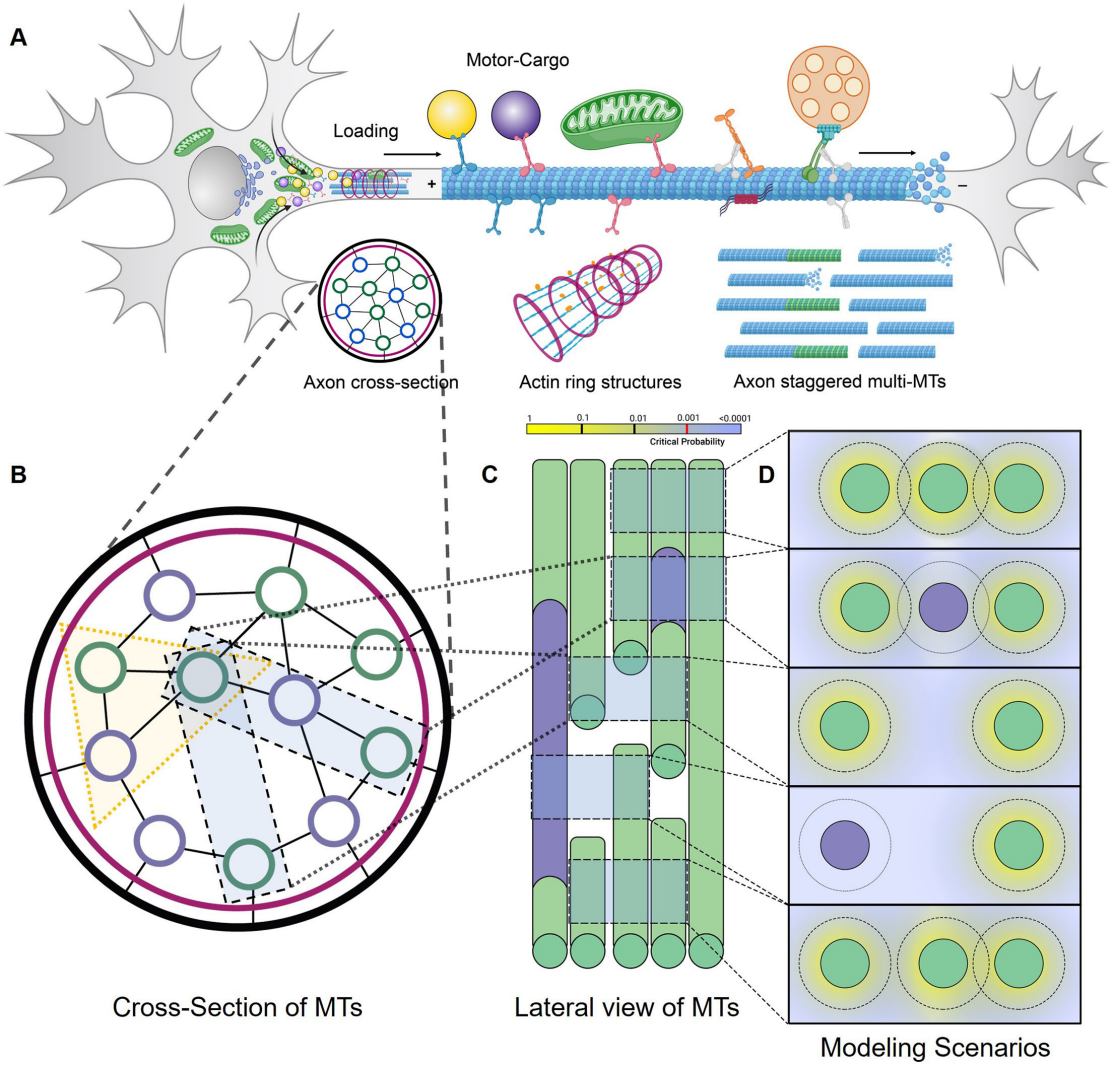
DyNAMO is a theoretical axon MT framework. **(A)** Diagram representation of a biological neuron, highlighting cargo-carrying kinesin transport on a single MT segment as part of a larger staggered multi-MT axon framework. Although MTs contain 13 protofilaments arranged in a cylindrical structure with a diameter of ~25 nanometers, DyNAMO models dual distinct kinesins along a single MT track/lane to restrain model complexity. Both axon superstructures (not modeled) and staggered MTs are implicated in influencing axonal transportation dynamics. **(B–D)** Illustrated in views are regions of gaps or full access to MTs, staggering in MT structures, and non-accessible regions [purple] that may arise as a consequence of damage or lost access due to bound proteins, tubulin code, or other mechanisms. **(B)** Axon cross-section slice is an end-on view of MTs. In DyNAMO, we model dual kinesin transport on a three-MT MMLS that allows us to simplify constraints while retaining sufficient model complexity. **(C)** Axon lateral slice view. **(D)** End in view of three-MT MMLS scenarios. The outer ring around MTs represents the modeling of a mathematical gradient probability distribution for the detachment and reattachment of a kinesin from MTs into inter-MT productive reservoirs. The motor lifetime in reservoirs is set at 1,000 ns which is non-limiting in our studies.

We developed a multiple MT lattice structure (MMLS) for axonal MT bundles that are designed on the basic framework of TASEP-LK, significantly modified to enable a nanoscale functional analysis of discontinuous staggered MTs with interacting dual kinesins and distinct kinesin-specific motility parameters in a theoretical axonal framework. Table 1 summarizes the experimental and defined parameters applied. Combined TASEP-LK has been applied by others to analyze unidirectional single motor flow in single MTs and motor particles for the co-existence of high- and low-density trafficking regions (Parmeggiani et al., 2003, 2004; Nishinari et al., 2005) or in two-MT models with motors as particles with symmetric or asymmetric distribution (Wang et al., 2007). In DyNAMO, the influx of the two kinesins into the MMLS at MT minus ends is regulatable and followed by stochastic progression of the kinesins along binding sites with lateral MT transitions permissible depending on MMLS scenarios. The only interactions considered between the two kinesins modeled in this study are those that result on a given single lane as hindered forward progression due to varied motility. To incorporate asymmetric coupling with MTs (Pronina and Kolomeisky, 2004, 2006; Jiang et al., 2007, 2008; Xiao et al., 2010), we used a three-MT MMLS and lateral movement parameters introduced for both kinesins to regulate crowding dynamics (Figure 1C). Two neuronal kinesins, Kin1 (S) and Kin3 (F) are described in the text, chosen for their significance and differences in motility and processivity (Verbrugge et al., 2009; Sun et al., 2011; Soppina et al., 2014; Lessard et al., 2019). LK detachment/reattachment dynamics (Jiang et al., 2007; Wang et al., 2007; Vuijk et al., 2015) are evaluated relative to kinesin-specific parameters and MMLS interactions. Detachment of kinesins into finite-length productive reservoirs at MT corresponding lattice sites in DyNAMO, instead of a singular bulk reservoir used in other studies, allows detailed retention of spatio-temporal dynamic information. The influx of kinesins into the MMLS is done via TASEP as bulk access to MTs and has measurable impacts on crowding and forward progression in coupled multilane interactions. Our DyNAMO model is the first theoretical axon model capable of capturing the nanoscale dynamics of multiple kinesins trafficking on a three-MT expandable TASEP-LK framework of discontinuous staggered MTs. DyNAMO expands the critical network of heuristic and mathematical analysis of compartmentalized sub-neuronal functions.

**Table 1:** Parametric values used in the simulations.

**Sl. No**	**Attributes**	**Symbol**	**Values**	**Units**	**Simulation equivalent**	**Experimental references**
1	No. of parallel MTs (one protofilament per MT considered)	D	3	In numbers	Subset of MTs per axon diameter Typical = 12–20 (Human = 13)	Ledbetter and Porter, 1964; Tilney et al., 1973; Sui and Downing, 2010
2	No. of MT segments (MMLS scenario conditions)	j	1 or 3	In numbers	Single or multi scenarios (1 or 3 segments)	Defined here
3	Length of each MT	L	4,000	Nanometer	Human MTs	Yu and Baas, 1994
					4.02 +/– 5.28 um	
4	Motor step size (l = L/N)	l	8	Nanometer	1 site	Svoboda et al., 1993; Kojima et al., 1997; Coy et al., 1999; Okada et al., 2003
5	Lattice sites on each MT (No. of 8 nm kinesin binding sites)	N	500	In numbers	Length dependent	Defined here
6	Loading rate of Kin3 (F) from the reservoir to MT lane	α_*a*_	10	Motors/s	2 motors in 10 timestamps	Defined here
			15	Motors/s	3 motors in 10 timestamps	Defined here
7	Loading rate of Kin1 (S) from the reservoir to MT lane	α_*b*_	10	Motors/s	2 motors in 10 timestamps	Defined here
			15	Motors/s	3 motors in 10 timestamps	Defined here
8	Outflow/delivery rate of Kin3 (F)	β_*a*_	5–8	Motors/s	1–2 motors in 10 timestamps	Simulated result
9	Outflow/delivery rate of Kin1 (S)	β_*b*_	5–8	Motors/s	1–2 motors in 10 timestamps	Simulated result
10	Motility rate Kin3 (F) (*v_*a*_*) [Human Kif1A]	*v*_*a*_	1,350	Nanometer/s	~7 steps per time *t*	Lessard et al., 2019
12	Motility rate Kin1 (S) (*v_*b*_*) [Human KHC]	*v*_*b*_	620	Nanometer/s	~3 steps per time *t*	Sun et al., 2011
13	Processivity of Kin3 (F) [Human Kif1A]	*p*_*a*_	6,240	Nanometer	~780 sites	Lessard et al., 2019
14	Processivity of Kin1 (S) [Human KHC]	*p*_*b*_	1,070	Nanometer	~130 sites	Verbrugge et al., 2009
15	Lifetime of kinesins in the productive reservoir	lt	60	Seconds	1,500 iterations (not limiting) up to 37 h	Brunner et al., 2004; Verbrugge et al., 2009
16	Minimum waiting time for ATP regeneration	wt	10	Seconds	250 iterations	Defined here
17	Productive reservoirs length and capacity	c [ ][ ]	400	Nanometer	Equivalent to sites; 50 motor capacity	Defined here
18	Observation time stamp	T	600	Seconds	15,000 iterations	Defined here
19	Iteration time stamp	t	40	Milliseconds	1 iteration	Defined here

### 2.2. Numerical model of the axonal MT architecture

To represent the complex multiple MT and staggered architecture in the axon we extend a simple TASEP single MT framework, applying basic MT descriptive principles of a lattice with sites. According to Parmeggiani et al. (2004), a one-dimensional TASEP was used, in which the analytical solution applied mean-field approximation to the Heisenberg equation and compared outcomes with Monte Carlo simulation results. Similarly, we defined a finite one-dimensional lattice with sites labeled *i* = *1, 2, … N* with lattice spacing *l* (8 nm). The stochastic equation for the bulk lattice sites (1 ≤ *i* ≤ *N*−1) with attachment (A) and detachment (D) dynamics of an MT is described by a master equation, where $\frac{d\langle n_{l}\rangle }{dt}\;$determines the rate of change in expected occupancy of a site and 〈 〉 characterizes the statistical average.


(1) $$\frac{d\langle n_{l}\rangle }{dt}=\;\langle n_{l-1}\left(1-n_{l}\right)\rangle -\langle n_{l}\left(1-n_{l+1}\right)\rangle \;+\;\omega_{A}\langle 1\;-\;n_{l}\rangle \;-\omega_{D}\langle n_{l}\rangle$$

The subsequent work by Verma et al. (2015) and Verma and Gupta (2018) resulted in a heuristic designed for analysis of a single MT with two lanes. In DyNAMO the tracks represent *MT protofilaments* on MTs in fully asymmetric coupled TASEP-LK under open boundary conditions. The evolution of motor density in the bulk for two lanes was given by:


(2) $$\begin{array}{l} \frac{d\langle n_{1}^{i}\rangle }{dt}=\omega_{A}\langle 1\;-\;n_{1}^{i}\rangle \;+\;\langle n_{1}^{i-1}(1-\;n_{1}^{i})\rangle -\langle n_{1}^{i}(1-\;n_{1}^{i+1})\rangle \\ \;-\omega_{D}\langle n_{1}^{i}\rangle \;-\;\omega \;\langle n_{1}^{i}n_{1}^{i+1}(1-\;n_{2}^{i})\rangle \end{array}$$

(3) $$\begin{array}{l} \frac{d\langle n_{2}^{i}\rangle }{dt}=\omega_{A}\langle 1\;-\;n_{2}^{i}\rangle \;+\;\langle n_{2}^{i-1}(1-\;n_{2}^{i})\rangle -\langle n_{2}^{i}(1-\;n_{2}^{i+1})\rangle \\ \;-\omega_{D}\langle n_{2}^{i}\rangle \;-\;\omega \;\langle n_{1}^{i}n_{1}^{i+1}(1-\;n_{2}^{i})\rangle \end{array}$$

The positive and negative terms on the right-hand side denote gain and loss terms arising due to kinesin attachment, detachment, and forward hopping succeeded by lane-changing processes. At the boundaries, particle densities are given by:


(4) $$\frac{d\langle n_{t}^{1}\rangle }{dt}=\;\alpha_{A}\langle 1-\;n_{t}^{1}\rangle \;-\langle n_{t}^{1}(1\;-\;n_{t}^{2})\rangle$$

(5) $$\frac{d\langle n_{t}^{L}\rangle }{dt}=\;\langle n_{t}^{L-1}(1\;-\;n_{t}^{L})\rangle \;-\;\beta_{A}\langle n_{t}^{L}\rangle$$

### 2.3. Capturing individual motor variables in a combined system of multiple motors

To capture kinesin motility parameters along with any inter-dependencies of transport and processivity, we combined multiple parameters as step-dependent equations. The dependency on multiple motor parameters creates a challenge to incorporate all variables into solvable partial differential equations (PDEs). To be able to capture individual motor variables in a combined system of multiple motors, as well as any interdependencies, we combine multiple motility parameters of speed and processivity into the following step-dependent equations. We let $\langle n_{t}^{i}\rangle \;$and $\langle m_{t}^{i}\rangle$ denote the discrete binary states of occupancy of protofilament lattice sites by motor species “a” and “b” representing kinesins at site “*i*” in lane *t*, respectively. The forward hopping rate or step size of species “a” is normalized to be a unit, and the forward hopping rate or step size of “b” is assumed *v* ≤ 1. The reattachment (R) rate of species “a” and “b” to any unoccupied lattice site *i* is denoted by ω_*a, R*_ *and ω*_*b, R*_, and the detachment (D) rate from the lattice site is denoted by ω_*a, D*_ *and ω*_*b, D*_. For sites 1 ≤ *i* ≤ *N*−1, the time evolution of $n_{t}^{i}\;and\;m_{t}^{i}\;$are governed by the following PDEs to incorporate asymmetric coupling of motors in the lanes with TASEP-LK and limited processive steps on lane *t*:


(6) $$\begin{array}{l} \frac{d\langle n_{t}^{i}\rangle }{dt}=\omega_{a,R}\langle 1-n_{t}^{i-1}n_{t}^{i}n_{t}^{i+1}-\;m_{t}^{i-1}m_{t}^{i}m_{t}^{i+1}\rangle \;-\omega_{a,D}\langle n_{t}^{i}\rangle \\ \;+\langle n_{t}^{i-1}\left(1-\;n_{t}^{i}-\;m_{t}^{i}\right)\rangle -\langle n_{t}^{i}\left(1-\;n_{t}^{i+1}-\;m_{t}^{i+1}\right)\rangle \\ \;-\;\omega_{a,l}\langle n_{t}^{i}n_{t}^{i+1}\left(1-\;n_{t-1}^{i}-\;m_{t-1}^{i}\right)\left(1-\;n_{t+1}^{i}-m_{t+1}^{i}\right)\rangle \\ \;+\;\omega_{a,l}\langle n_{t-1}^{i}n_{t-1}^{i+1}\left(1-\;n_{t}^{i}-m_{t}^{i}\right)+\;n_{t+1}^{i}n_{t+1}^{i+1}\left(1-\;n_{t}^{i}-m_{t}^{i}\right)\rangle \end{array}$$

(7) $$\begin{array}{l} \frac{d\langle m_{t}^{i}\rangle }{dt}=\omega_{b,R}\langle 1-n_{t}^{i-1}n_{t}^{i}n_{t}^{i+1}-\;m_{t}^{i-1}m_{t}^{i}m_{t}^{i+1}\rangle \;-\omega_{b,D}\langle m_{t}^{i}\rangle \\ \;+\;\langle vm_{t}^{i-1}\left(1-\;n_{t}^{i}-m_{t}^{i}\right)\rangle -\langle vm_{t}^{i}\left(1-\;n_{t}^{i+1}-\;m_{t}^{i+1}\right)\rangle \\ \;-\;\omega_{b,l}\langle m_{t}^{i}m_{t}^{i+1}\left(1-\;n_{t-1}^{i}-\;m_{t-1}^{i}\right)\left(1-\;n_{t+1}^{i}-m_{t+1}^{i}\right)\rangle \\ \;+\;\omega_{b,l}\langle m_{t-1}^{i}m_{t-1}^{i+1}\left(1-\;n_{t}^{i}-m_{t}^{i}\right)+\;m_{t+1}^{i}m_{t+1}^{i+1}\left(1-\;n_{t}^{i}-m_{t}^{i}\right)\rangle \end{array}$$

The complex correlation between multiple motors in the MMLS model made it impossible to find an analytical or numerical solution using partial differential equations (PDEs) without removing these interdependencies. Furthermore, external parametric conditions such as the processivity and lifetime of motors added more complexity to the equation. Thus, a heuristic approach was used to solve MMLS, which involved defining specific testable algorithm conditions to examine these complex interactions. The forward hopping of motors and asymmetric coupling with adjacent lanes is governed by the TASEP principle but our model allowed for motors to make multiple hops with their respective speeds within one-time frame. The natural exit and entry of motors from/into the productive reservoir into/from the MT lanes are governed by the principles of LK. The entire dynamics of the motor are subdivided into four major action phases—loading, forward progression, detachment–reattachment, and output/delivery governed by the TASEP-LK principles with asymmetric coupling.

### 2.4. The influx of kinesins onto the minus ends of MT MMLS lanes

In DyNAMO, the finite junction reservoir loads Kin3 (F) and Kin1 (S) onto MT binding sites following a normalized Gaussian distribution. The inter-arrival time or the time between successive loading is distributed. The expected run time of the simulation is proportional to motor generation rate α_*a*_, α_*b*_ (“influx rate” in motors/s). In Scenarios 1–8, both Kin3 (F) and Kin1 (S) are loaded onto MTs one kinesin at a time from the finite productivity reservoir present at the minus end of the MT lanes. As described in Algorithm 1, kinesin loading is restricted to the binding site *i* = 1 (referred to as the loading site) at the minus end of each MT. The kinesins have access to all parallel lanes with equal probability in all scenarios, except when the flow is forcefully channelized to a single lane due to proximal staggering in Scenarios 3–5.

**Algorithm 1:** Kinesin influx model: loading of Kin3 (F) and Kin1 (S) on MTs.

0: **Input:** *Influx_rate (*α_*a*_, α_*b*_*), input_reservoir, particle, lane, site*
0: **Output:** *motor_cargo:* Total numbers of Kin3 (F) and Kin1 (S) motors attach
0: *Initialization*
0: An infinite reservoir of motor-cargo pair (*input_array*) is generated at the given generation rate (α_a_ and α_b_)
0: **for** *time*←1 **to** runtime **do**
0: temp←*input_reservoir* [*time*]
0: lane←one of the lanes (1/2/… T)
0: site←loading site (1)
0: particle [lane][site]←temp
0: **if** particle [lane][site] = = 1 **then** // motor_a (Kin3 (F)) is loaded
0: The other parameters (velocity, span, and lifetime) are updated accordingly.
0: **else if** particle [lane][site] = = 2 **then** // motor_b (Kin1 (S)) is loaded
0: The other parameters (velocity, span, and lifetime) are updated accordingly.
0: **else** // no motor is loaded
0: particle [lane][site] = 0
0: No changes.
0: **end**
0: **end**

### 2.5. Unidirectional traversal of kinesins along the MT lane (TASEP)

We confined our MMLS to a two-dimensional plane, which is necessary to simplify the current simulation to implement greater overall complexity with additional parameters and realize that this may create some bias (Whitesides, 1970). In our two-kinesin DyNAMO trafficking model, we have defined multiple one-dimensional LS of T lanes (T ≥ 2) of length *L* consisting of *N* lattice sites (*N* ≥ 3). The multiple one-dimensional binding sites are labeled *i* = *1, 2, 3 ... N*, and lattice spacing is *l*=*L/N*. The binding sites *i* = 1 and *i* = N define the left and right boundary, while *i* =2, 3 … *N*-1 is referred to as the bulk. The binding site is characterized by $\left\{n_{t=1,2,3\;\ldots \;t}^{i=1,\;2,\;3\;\ldots \;N}\right\}$, when the occupied state by motor “a” (denoted by $n_{t}^{i}$) or motor “b” (denoted by $m_{t}^{i}$) is equal to zero (vacant) or one (occupied). Every site can be occupied by either kinesin or not occupied by either kinesin type.

To model the transport of two different types of motors, the faster Kin3 (F) can traverse to the next open site at a rate of *v*_*a*_ *steps*, while the slower Kin1 (S) can traverse at a rate of *v*_*b*_ *steps* (assuming: *v*_*a*_ ≥ *v*_*b*_). No kinesin can hop over another to occupy a forward available site during traversing along the lane (protofilament). Kin3 (F) upon encountering a barrier (on the occupancy of the next site by Kin1 (S) or blockage), either laterally moves into an available lateral MT lane or gets detached into the productive reservoir. Kin1 (S) is a limited processivity motor. To characterize the processivity component of motility, we implement Kin1 (S) with a processivity length (the maximum distance the motor traverses on an MT before detachment) of 1,070 nm (i.e., equivalent to ~130 lattice sites). The progressive system dynamics for different MT transitions based on the modified TASEP are governed by the following methodologies:

1. **Loading:** At the site (*i* = *1*), a motor can enter the MMLS with rate α if unoccupied.
2. **Forward progression:** If the sites (*i*+*1, i*+*2 … i*+*v)* are unoccupied, a motor with velocity v can jump from site (*i*) to site (*i*+*v*) that is equivalent to an 8 nm dimeric kinesin step in DyNAMO.
3. **Lateral movement:** If any of the sites (*i*+*1, i*+*2 … i*+*v)* are occupied, a motor can jump from site (*i*) to either site (*i-1*) or *(i*+*1)* of an adjacent lane if unoccupied.
4. **Delivery/output:** At the site (*i* = *N-v*+*1, N-v*+*2 … N)*, if a motor with velocity v is present it can leave the MMLS from the plus end of the MT lane.

Limited processivity requires the movement of kinesins to detach from MTs into an inter-MT reservoir from which they can either reattach onto the same MT or an adjacent MT in the MMLS. When congestion prevents reattachment to the same MT, reattachment must occur to an adjacent MT or the kinesin is retained in the productive reservoir until sites are available. A general Algorithm 2 governing the TASEP model is formulated on the basis of the above methodologies. Different simulation paradigms are considered for different scenarios. For example, in Scenario 2, we limit the provisions for lateral movement; whereas in all other scenarios, both Kin3 (F) and Kin1 (S) have provisions to move to adjacent MTs upon encountering congestion. In Scenarios 3–8, a portion of MMLS is staggered to channel kinesin flow into a singular MT lane either in proximal or distal ends to evaluate blocked access by a variety of mechanisms (damage, tubulin modifications, MAPs, and so forth) as described in the text. The kinesins can overcome crowding by detaching and reattaching to the localized productive reservoir and MT availability for movement to an adjacent lateral MT. Previous studies have included similar concepts of MT lanes and lattice sites (Dixit et al., 2008; Che et al., 2016; Liang et al., 2016), productive reservoirs (Leduc et al., 2012; Ciandrini et al., 2014; Feng et al., 2018), or side-stepping lateral movement to an adjacent MT lane (Wang et al., 2007; Jiang et al., 2008; Hoeprich et al., 2014), which are also conceptually included in DyNAMO.

**Algorithm 2:** TASEP model: forward traversal of Kin3 (F) and Kin1 (S) on MTs.

0: **Data:** *particle, lane, site*
0: *Initialization*
0: **for** *time*←1 **to** runtime **do**
0: **for** *i*←1 **to** T and *j*←1 **to** binding sites **do**
0: gap←distance between two motors
0: lane←i;
0: site←j;
0: **if** *gap* ≥ *velocity* **then** // motor moves with constant velocity
0: Motor **jumps** from the site (*j*) → (*j*+*velocity*)
0: The other parameters (velocity, span, and lifetime) are updated accordingly.
0: **else if** *gap*<*velocity* **then** // lateral movement of motor
0: **if** the *corresponding (j*+*velocity) site of adjacent lanes is empty* **then**,
0: Motor **jumps** to this site of adjacent lanes *i* ± 1
0: The other parameters (velocity, span, and lifetime) are updated accordingly. //crowding-induced detachment of motor
0: **else if** the *corresponding (j*+*velocity) site of adjacent lanes is blocked due to staggering* **then**,
0: Motor is **detached** into the productive reservoir.
0: Particle [lane][site]←0
0: **end**
0: **end**
0: **if** *no. of sites traveled on microtubular lane* ≥ *processivity* **then** //processivity-induced detachment
0: motors are **detached** into a productive reservoir.
0: Particle [lane][site]←0
0: **end**
0: **end**
0: **end**

### 2.6. Reattachment and detachment dynamics into productive reservoirs (LK)

Productive reservoirs are defined by LK from the lattice sites (1 ≤ i ≤ N-1) of the MMLS. In previous studies, TASEP-LK (Wang et al., 2007; Vuijk et al., 2015; Dhiman and Gupta, 2016) was used to define the reattachment and detachment of kinesins to and from a bulk productive reservoir. In DyNAMO, we define productive reservoirs instead as a queue of a fixed size corresponding to each lattice site (motor binding site) on MTs of the MMLS. The LK dynamics of reattachment and detachment are defined accordingly:

1. **Detachment:** During the kinesin motility along one-dimensional lattice sites, detachment occurs when one motor cannot take the assigned hops (stepping) due to crowding. That is, if the site (*i*+*1, i*+*2 … i*+*v)* of the MT lanes, such as Kin3 (F) trailing Kin1 (S), will result in Kin3 (F) being detached into a productive reservoir at lattice site i.
2. **Reattachment:** In the productive reservoir at lattice site i, within a given lifetime of the motor, if a site (*i-1, i, i*+*1*) is unoccupied, then the motor reattaches to an open site on the MT.
3. **Leakage:** If a motor does not reattach within its lifetime, then we describe the motor as leaked out of the system into a non-productive reservoir.

Algorithm 3 describes the process of reattachment/detachment of motors (also described as absorption and desorption). We apply LK to describe the off–on dynamics of motors along the MTs because of crowding, which also creates opportunities for motors to reattach to lateral MTs to avoid local crowding along MTs. All our scenarios considered for simulation have provision for kinesin reattachment back to MTs on the availability of vacant sites corresponding to the point of detachment. Scenarios 1 and 2 featured parallel MT lanes, while Scenarios 3–8 included staggering to limit access to adjacent MTs, either proximally at the minus end or distally at the plus end of MTs. The LK model enabled the interpretation of the on–off dynamics of kinesins under different scenarios and flow rates.

**Algorithm 3:** LK model: process of reattachment and detachment of Kin3 (F) and Kin1 (S) to and from the productive reservoir along the MTs.

0: **Input:** *particle, productive_reservoir, wait, leakage, lifetime, lane, site*
0: **Output:** *wait:* Number of Kin3 (F) and Kin1 (S) waiting in productive reservoir for reattachment
0: *leakage:* Number of Kin3 (F) and Kin1 (S) detached out of the system in a non-productive reservoir
0: *Initialization*
0: Productive reservoir of queue length (fixed) and non-productive reservoirs is present at each corresponding site of the microtubular lane.
0: **for** *time*←1 **to** runtime **do**
0: **for** *i*←1 **to** T and *j*←1 **to** N **do**
0: lane←i;
0: site←j;
0: **if** *lifespan of motors in productive_reservoir(c*[*lane*] [*site*]) ≥ lifetime(lt) **then** // leakage of motor
0: Motor is popped from the productive reservoir and pushed to the non-productive reservoir.
0: leakage←leakage+1
0: **else if** *lifespan of motors in productive_ reservoir(c*[*lane*][*site*]) < lifetime(lt) **then** // reattachment
0: Motor is popped from the productive reservoir and reattached to (1 of 9) neighboring sites of corresponding_particle [lane][site].
0: The other parameters (velocity, span, and lifetime) are updated accordingly.
0: **end**
0: **end**
0: **end**

### 2.7. Delivery of kinesins from the MT plus end

The transit of Kin3 (F) and Kin1 (S) is to the distal plus end (*i* = N) of an MMLS by motor service rate β_*a*_, β_*b*_ given as an “Outflow rate” in motors/s). At the end of an MT lane, the kinesins are transferred to a junction reservoir (described in Algorithm 4). These kinesins at the junction reservoir form a limited pool of available kinesins that can be loaded onto the next MMLS segment. This limited reservoir of kinesins is analyzed in the evaluations of mixed organization of MMLS scenarios in an extended axonal framework.

**Algorithm 4:** Kinesin delivery model: process of delivery of Kin3 (F) and Kin1 (S) at MT plus ends to a junction reservoir.

0: **Input:** *particle, velocity, throughput, lane, site*
0: **Output:** *wait:* Number of Kin3 (F) and Kin1 (S) delivered from plus end
0: *Initialization*
0: **for** *time*←1 **to** total_simulation_time **do**
0: **for** *i*←1 **to** T and *j*←1 **to** lattice sites **do**
0: lane←i
0: site←j
0: **if** *j*+*velocity* ≥ *N (total_lattice_site)* **then** // delivery of motor
0: motor jumps from site (*j*) → junction_repair
0: *particle*[*lane*][*site*]←0
0: *throughput*←*throughput*+1
0: **end**
0: **end**
0: **end**

### 2.8. Solution scheme paradigms

The simulation environment uses Python 3.8 for testing the algorithms in DyNAMO. The code and documentation developed for DyNAMO are available in the open platform GitHub. The parameters used are in accordance with biologically observed data obtained from numerous different sources and summarized in Table 1. In summary, the model starts by setting up the MMLS system of T number of parallel lanes and N binding sites. The dual-motor dynamicity is parameterized by their individual speed/velocity (v), processive steps (p), and influx rate (α). Based on the chosen scenario, the MMLS is set up with different boundary conditions and restrictions within the system. The kinesins from the finite reservoir in the minus end are loaded onto MT initial sites at each iterative time stamp. The motors based on the different decisions governed by TASEP and LK, gradually traverse along MTs and exit the system from MT plus ends. The evaluated metrics (such as outflow rate, dissociation, and dynamicity) and critical condition cases are considered for depicting different kinesins of distinct parameters trafficking across various scenarios. To extensively study the effect of these parameters on multiple linked segments of MMLS scenarios, we considered a linked architecture with varied linked MMLS having different scenario restrictions. These individual MMLS segments evaluated in a multiple MMLS scenario retain their outcomes as discrete segments but when linked demonstrate the broader impact of distributed or concentrated MT injuries. The simulations were run on standard desktop processors (Intel i7-9th generation with RTX 2080 Ti) and took ~3–4 h for each scenario. The subcases of each scenario were also evaluated on GPU clusters at Purdue University. In future, the DyNAMO model can be scaled up to analyze longer MT tracks with more protofilaments per MT, as well as incorporate additional kinesins. However, such an increase in variables will significantly increase the computational complexity of the model and require access to more powerful computing resources such as larger clusters or supercomputers.

## 3. Results

### 3.1. DyNAMO, a parameter-dense theoretical axon framework

To evaluate combined contributions of multiple kinesins on a disease- or injury- perturbed axonal MT infrastructure requires the ability to capture detail-rich dynamic information. In DyNAMO, we apply a TASEP-LK paradigm approach for non-equilibrium and equilibrium statistical mechanics, summarized here and detailed in Methods. The simulations are analyzed in the environment of Python 3.8, with parametric values as defined from experimental measurements or by assumptions based on published experimental data (Table 1). Two unidirectionally trafficking kinesins with different motility and processivity rates are mathematically described to enable considerations of crowding, including influx rates, and the influence of motor type in damage scenarios. In this study, we focus on human conventional Kinesin-1 KHC and Kinesin-3 Kif1A neuronal kinesins referred to herein as Kin1 (S) and Kin3 (F) slower and faster motors. Electron microscopy studies of longitudinal or transverse axon sections were used to provide guidance on parameters for MT discontinuous organization. In human hippocampal developing neurons, shorter MT lengths are reported (4.02 +/– 5.28 um; Yu and Baas, 1994). Detailed MT cytoskeleton maps along axon length in *Caenorhabditis elegans* neurons are generally slightly longer 1.2–10.7 um in length (Chalfie and Thomson, 1979). We apply a three-MT framework MMLS during simulations with the ability of kinesins to move between all MTs and capture features of normal or increased staggering as a simulation of MT injury (Matamoros and Baas, 2016; Figure 1). The MT surface is modeled as a single lattice structure, single protofilament-like, with N kinesin-binding sites along a defined but adjustable MT length. Axonal MTs are non-contiguous staggered short segments (Figure 1A). Maximum MT lengths for this study are set at 4,000 nm, reflective of average MT segment lengths observed experimentally (Yu and Baas, 1994), and containing up to 500 binding sites of stochastically Gaussian-envelope-like kinesin steps of 8 nm, the step size of dimeric kinesin moving along a protofilament (Svoboda et al., 1993; Kojima et al., 1997; Coy et al., 1999; Okada et al., 2003).

In TASEP, motor interactions with MTs are described by hard-core repulsion, such that each binding site is accessible to only one of any kinesin at any moment. TASEP describes bulk kinesins entering or leaving the system at system boundaries (MT ends), such as from the AIS at the neuron soma (Petersen et al., 2014; Kapitein and Hoogenraad, 2015; Leterrier, 2016) or in interconnected multiple MMLS along the axon length. LK allows specified actions on transiting kinesins to be mathematically described, such as kinesins undergoing MT-to-MT jumps or dissociating into and reassociation from position-correlated inter-MT reservoirs. Detachments can occur via internal crowding, processivity limitations, or termination of MT segments, with MT reassociation when there is open access. The use of positionally distinct inter-MT reservoirs in DyNAMO rather than a long bulk reservoir (Leduc et al., 2012; Feng et al., 2018) allows us to capture stationary positional with on/off dynamicity. High dynamicity reflects crowding and highlights delayed forward progression points along the theoretical axon. The positional reservoirs have an adjustable storage capacity that can be restricted in capacity and time. Kin1 (S) has been observed to have a long but definitive lifespan of ~37 h in the vicinity of axonal MTs (Brunner et al., 2004; Verbrugge et al., 2009), and in our shorter-term scenarios, no motors are lost from inter-MT reservoirs, and all eventually return to the system. The full simulation environment is detailed in Methods. The corresponding kinetic rates obey a detailed balance such that the system evolves into an equilibrium steady state.

### 3.2. Initial patterning and progression of kinesins on the theoretical axon

**Figure 2:**
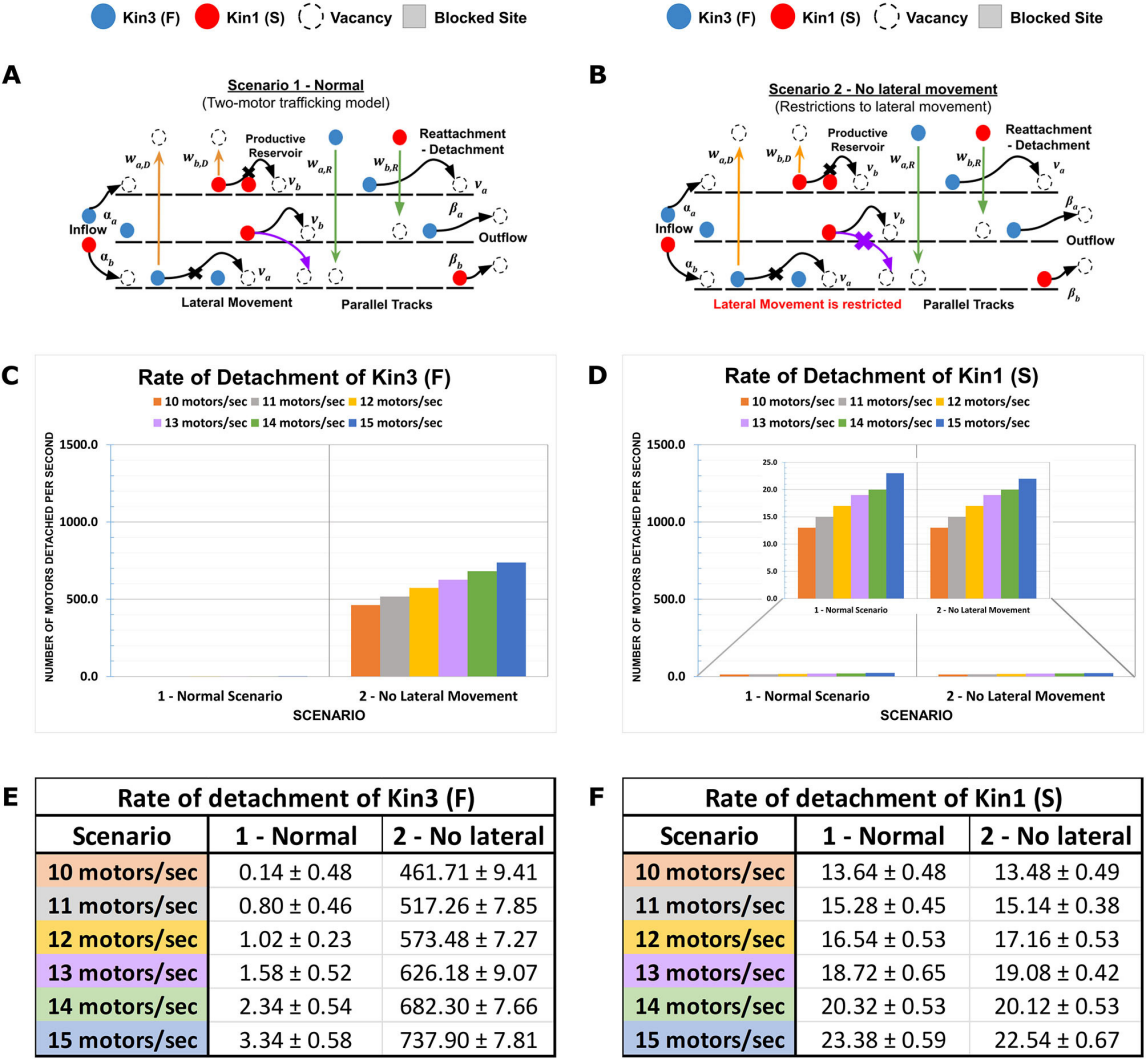
Contributions of Influx rate and lateral MT-to-MT access in defining axonal kinesin progression. Traversing kinesins reflect an overall dynamic that involves influx, forward motion, detachment, and reattachment to the same or an adjacent parallel MT in the MMLS based on site availability. **(A, B)** Two MMLS scenarios are depicted, one with provision (Scenario 1) for kinesins to move laterally onto adjacent parallel lanes and one without (Scenario 2). **(C, D)** Detachment metrics of Kin1 (S) and Kin3 (F) are measured and plotted in histograms in relation to influx rate and MMLS scenarios for parallel MT lanes. **(E, F)** In Scenario 1, accessibility to parallel MTs is reflected in the low detachment of Kin1 (S) and Kin3 (F). However, in Scenario 2, restricted lateral movement along with an increase in influx rates promotes the detachment of faster motor due to hindrance (10x-15x for influx rates of 10–15 motors/s).

In previous studies applying TASEP to two-lane systems, single particle dynamics vary due to exchange between lanes at asymmetric rates (Pronina and Kolomeisky, 2006; Jiang et al., 2007, 2008; Cook et al., 2009; Vuijk et al., 2015; Dhiman and Gupta, 2016; Gupta, 2016). Using DyNAMO, we evaluated the impact of “influx rate” in our multi-kinesin MMLS to study how kinesin-specific motility parameters such as speed and processivity, as well as MT-to-MT lateral access or restriction, impact progression. Two MMLS scenarios and outcomes are described in Figure 2. In MMLS Scenario 1 (Figure 2A), all MTs are accessible with lateral movement at any position. This allowance accommodates the presence of Kin1 (S) and Kin3 (F) of variable speed by allowing Kin3 (F) to jump laterally to adjacent MTs to avoid crowding and support forward progression. In Scenario 2 (Figure 2B), no lateral access is allowed. When the Influx rate is varied incrementally from 10 to 15 motors/s, Kin1 (S) is minimally affected, whereas the impact on the detachment of Kin3 (F) is significant with 400–700 motors detached per second. Comparative histograms (Figure 2C vs. Figure 2D) and quantitative analysis (Figure 2E vs. Figure 2F) are shown. The primary contributing factor to the detachment of Kin3 (F) is crowding, whereas, for Kin1 (S), it is limited processivity, which is consistent with previous theoretical models (Leduc et al., 2012; Ciandrini et al., 2014). In our analysis, early lateral access to MTs in axons has the primary impact on defining the initial kinesin traffic patterning in a multiple motor system, which is consistent with experimental observations *in vivo* (Rank and Frey, 2018).

### 3.3. Simulated axonal injury differently impacts kinesin types in dissociation or progression

**Figure 3:**
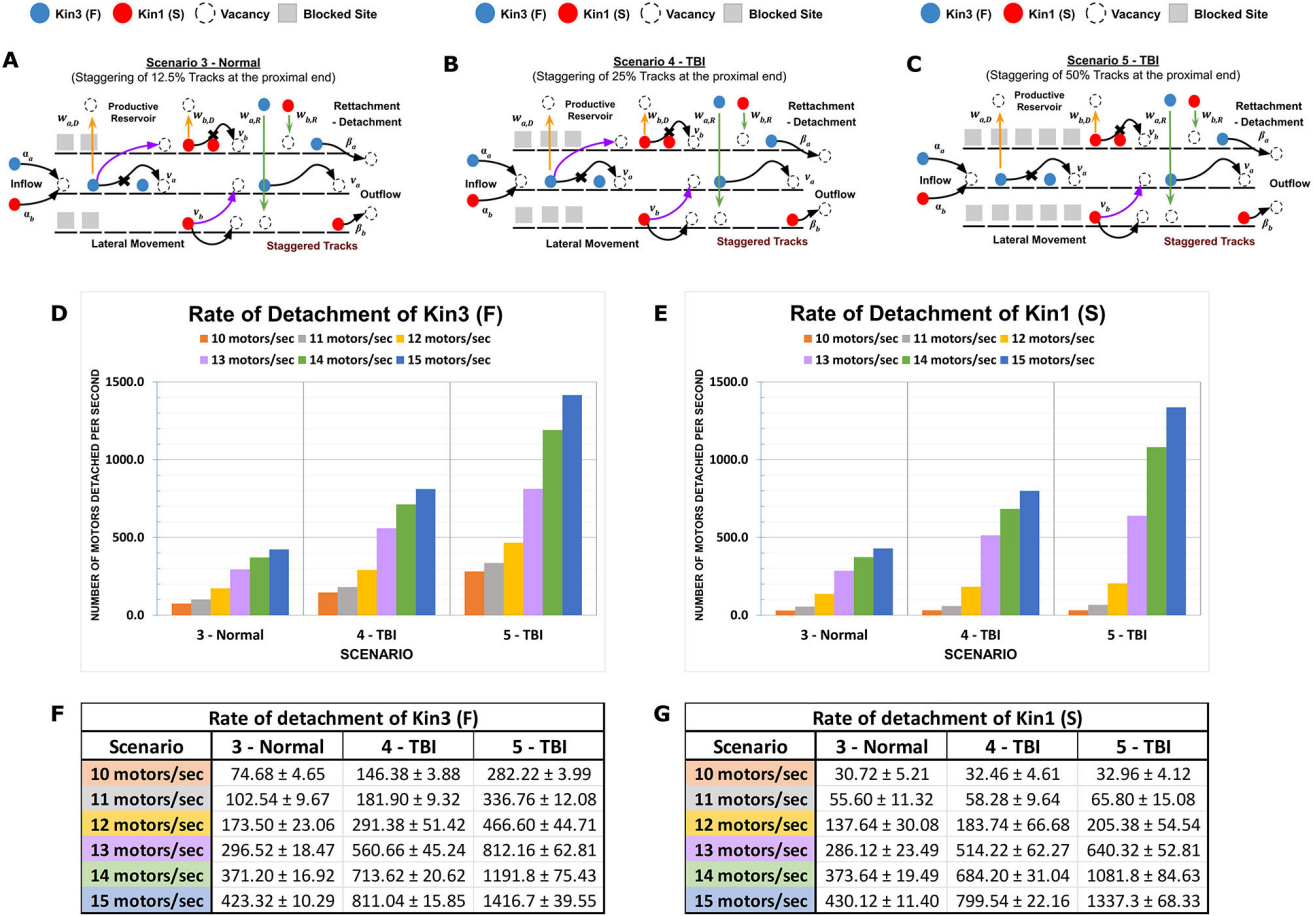
Proximal MT staggering on the channeling of kinesins. Kinesin speed (motility rate) and duration of movement (processivity) with multi-MT architecture combine to affect kinesin axonal progression. **(A–C)** Diagrammatic representations of MMLS scenarios for proximally staggered MTs. Staggering 12.5% (Scenario 3, Normal), 25% (Scenario 4, TBI), and 50% (Scenario 5, TBI) were modeled and are regions inaccessible to the kinesins. Higher staggering in Scenario 5 is considered a more severe injury to MTs. **(D, E)** A graph is presented that shows the effects of different influx rates on the detachment rates of Kin1 (S) and Kin3 (F) in staggered scenarios. The detachment rate for each motor is plotted on the y-axis, and the influx rate is plotted on the x-axis. **(F, G)** The channelization of the Kin1 (S) and Kin3 (F) from the proximal minus end on the MT lanes favors the flow of a kinesin with slower motility. At low influx rates (10–13 motors/s), a comparable difference in the detachment rates of Kin3 (F) with the Kin1 (S) is observed. However, beyond an influx threshold (14–15 motors/s), the increased motor density results in the channelization with increased rates of kinesins being forced into dynamic productive reservoirs.

To simulate axonal injury such as stretch-induced gaps or destabilization of MTs affecting length due to partial depolymerization, we modeled the impact of altered MT staggered lengths in our MMLS on kinesin progression vs. on/off dynamicity. Proximal or distal staggering was evaluated in multiple MMLS scenarios with 12.5% staggering considered within normal variation (Scenario 3) and 25–50% staggering reflecting increasing degrees of injury-induced perturbation (Scenarios 4 and 5, respectively). Staggering creates channelization of kinesins (Figures 3A–C). When staggering is proximal and the influx rate is low (< 13 motors/s), Kin3 (F) is impacted, whereas Kin1 (S) is not impacted (Figures 3D, E). However, at higher influx rates (>13 motors/s), bulk crowding occurs due to the channelization of multiple kinesin types with differing motility parameters onto a single MT lane. This results in a high rate of detachment of both Kin1 (S) and Kin3 (F) into the inter-MT reservoir. The detachment rate for staggered injured MT tracks (such as Scenarios 4 and 5) increases by 3–4 times compared to a normal MT Scenario 3 for faster Kin3 (F), while it results in an almost 10-fold increase for the slower Kin1(S). In addition, channelization and influx rates combine to limit the availability of free MT sites for kinesins to move along a singular MT lane. Thus, a huge dynamic on-off activity is observed with an increased length of channelization. DyNAMO reveals that during proximal channelization, and dependent on influx rate, the slowest moving kinesin dictates the progression of all the kinesins (see Figures 3F, G) until additional parallel MT lanes become available for MT-to-MT jumps. The outcomes support a mechanistic need to limit staggering within the AIS or apply gating controls at the AIS that have been proposed biologically (Petersen et al., 2014; Kapitein and Hoogenraad, 2015; Leterrier, 2016).

**Figure 4:**
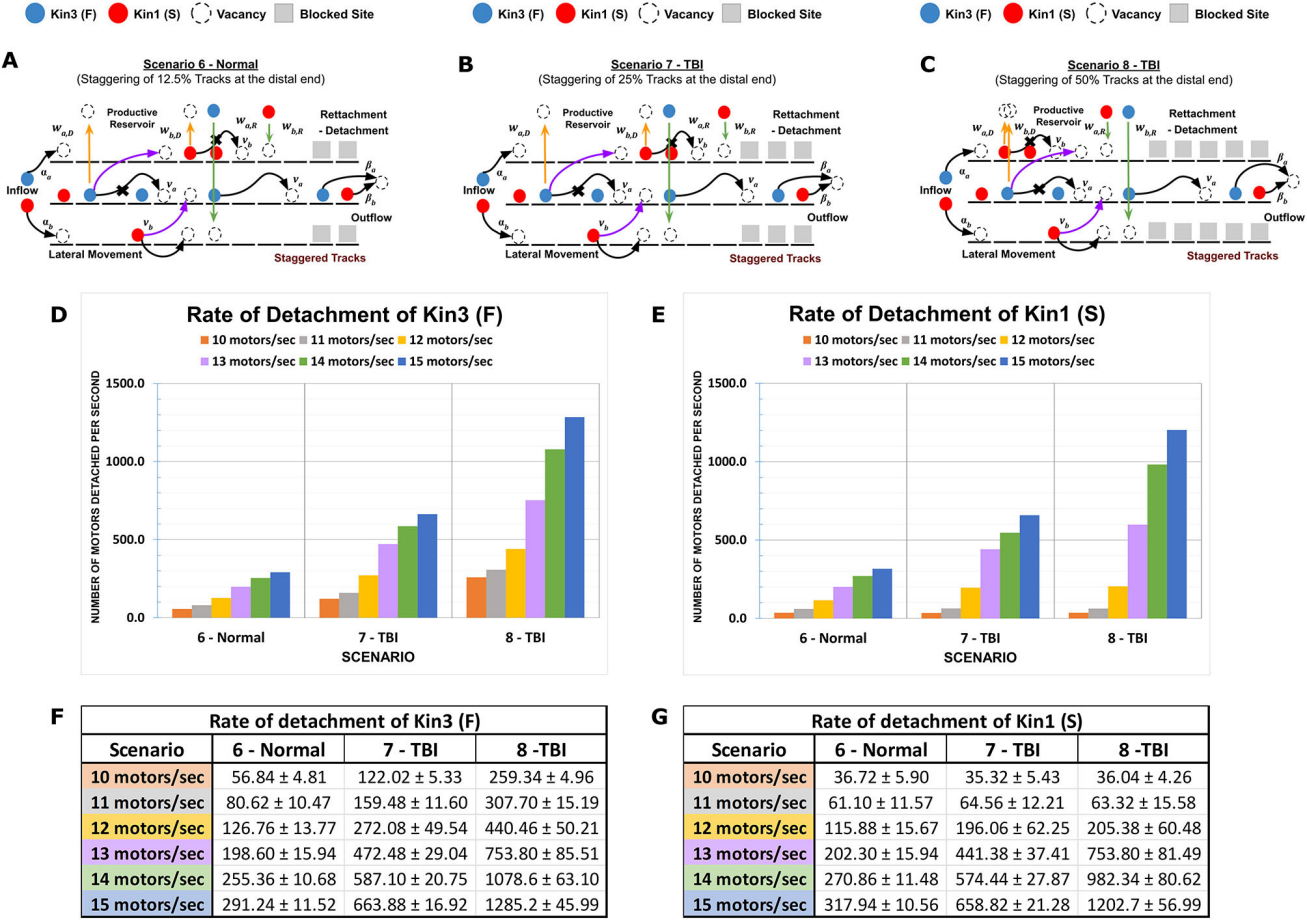
Distal MT staggering on the channeling of kinesins. Channelization of motors at the distal end of MTs has a considerable effect on kinesins and controls the outflow of motors from the plus end. Staggering at the distal end of adjacent MT lanes forces the freely flowing motors from parallel adjacent lanes onto a reduced number of MTs inducing crowding between Kin1 (S) and Kin3 (F) and motor detachment. **(A–C)** Diagrammatic representations of MMLS scenarios for distal staggered MTs of 12.5% (Scenario 6, Normal), 25% (Scenario 7, TBI), and 50% (Scenario 8, TBI). **(D, E)** Graphical representation showing Kin1 (S) and Kin3 (F) detachment rates for different influx rates for distal staggered scenarios. **(F, G)** Influx of kinesins at the point of transition to a single staggered path (channelization) creates immediate dense crowding of motors. This junction point results in the detachment/reattachment dynamicity of both Kin1 (S) and Kin3 (F) with productive reservoirs. At low influx rates, dynamicity is primarily influenced by motor processivity. However, at high influx rates, crowding plays a more dominant role in regulating the dynamicity of motors.

Increased staggering length at the distal end of the MMLS (Figures 4A–C) was also modeled at 12.5% (Scenario 6), 25% (Scenario 7), and 50% (Scenario 8). In distal staggering, the initial uniform loading and distribution of the motors along the parallel MT lanes abruptly transitions to channeling. Kin1 (S) and Kin3 (F) motors become channelized onto a dedicated single MT lane. At a low influx rate, Kin3 (F) is primarily affected, whereas a higher density of motors results in bulk crowding that impacts both Kin1 (S) and Kin3 (F) forcing increased detachment. Histogram plots of staggered vs. open MT regions (Figures 4D, E) show the effect of distal channelization on kinesins with different motility rates. In summary, an increased initial influx rate of motors from 10 to 15 motors/s results in denser packing of motors on the MMLS that increases dynamicity abruptly at the junctional change to staggering. The channelization length (12.5, 25, or 50% blockage of lateral binding sites) impacts the progression rate, defined by the slowest motor, and further impacts the degree of dynamicity along the staggered column (Figures 4F, G). The rate of on–off activity increases proportionately with increases in the length of staggered columns. The DyNAMO model reveals the importance of mapping the natural and injury distribution of staggered axonal MTs along the axon length in future. Furthermore, DyNAMO reveals that proximal staggering of axonal MTs at the AIS or along the axonal MT length can generate a gatekeeper effect dominated by slower kinesins, whereas distal staggering has a clearing effect that can remove the bulk of kinesins such that their reattachment will immediately influence the downstream distribution of kinesins on the theoretical axon and therefore signaling output.

### 3.4. Kinesin dynamicity is elevated in axonal injury and impacts concentrations of axon-distributed motors

**Figure 5:**
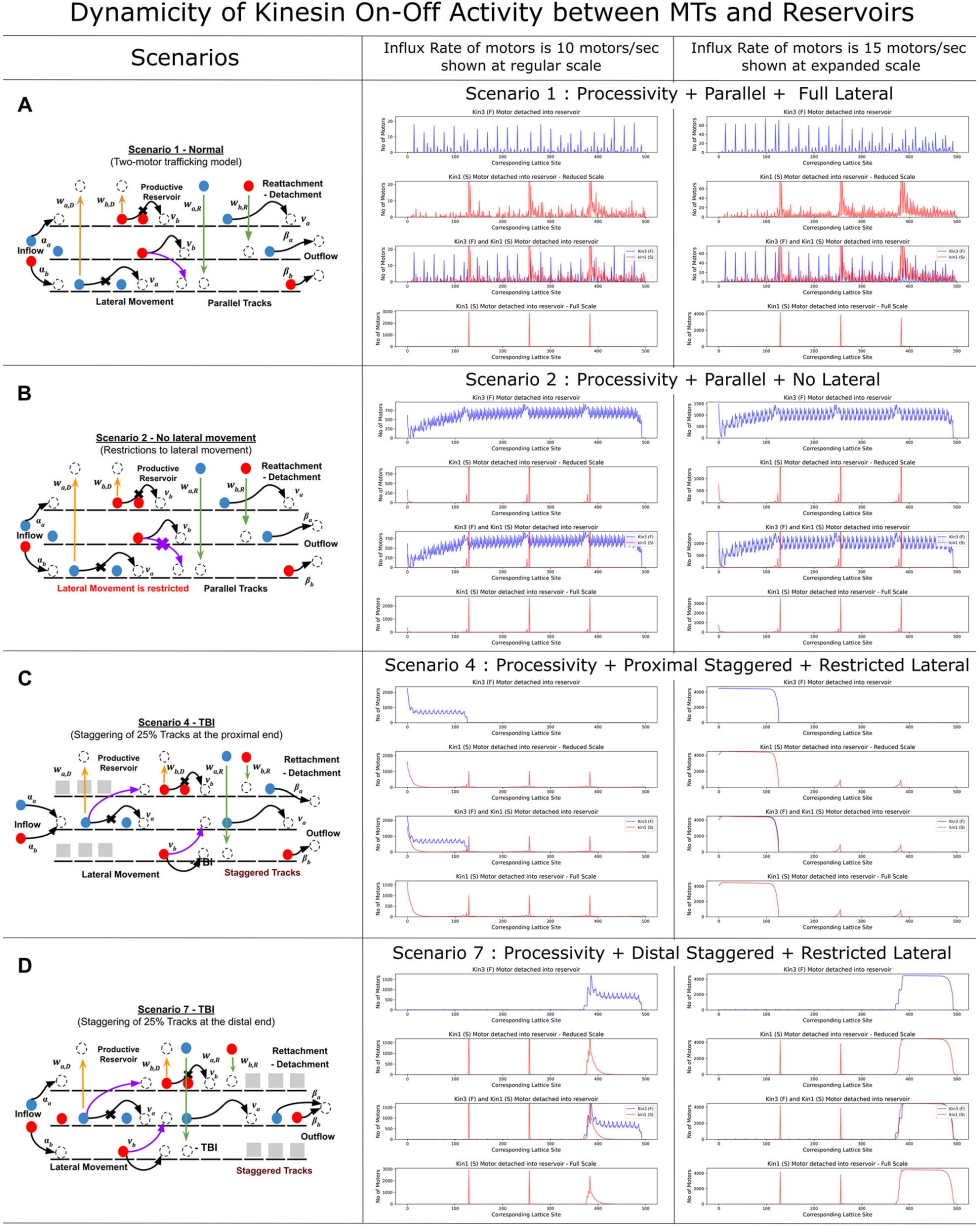
Axonal injury scenarios reveal increased kinesin dynamicity in lieu of progression. Influx rate, kinesin-specific parameters of speed and processivity, and crowding dynamics contribute to motor dynamicity that stalls the progressive flow of kinesins. Each graph represents the dynamicity of kinesin on-off activity with the productive reservoir. The first subplot shows the dynamicity of Kin3 (F) [Red] followed by a reduced scale flow of Kin1 (S) [Blue]. The next subplot shows a comparative study of both the kinesin [note the reduced axis]. The final subplot shows the detachment dynamics of Kin1 (S) in true scale. **(A)** For Scenario 1, Kin3 (F) and Kin1 (S) have a minimal collision throughout the lane, while the majority of the detachment of Kin1 (S) is limited by its processivity. **(B)** With the restriction of lateral movement in Scenario 2, the Kin3 (F) are heavily crowded by the slow-moving Kin1 (S) throughout the MTs, observed as the wide purple lines. **(C, D)** For staggered scenarios, a combination of channeling and higher influx rates results in significant changes in the flow and patterning of the kinesins on MTs.

We refer to the detachment and reassociation of kinesin as dynamicity and compare different scenarios of MT lattice staggering in the MMLS and influx rates on the dynamicity of kinesin types (Figure 5). The DyNAMO allows kinesin reattachment from reservoirs to MTs at plus/minus one step around that detachment site. Four MMLS scenarios with influx rates of 10 or 15 motors/s were compared (Figures 5A–D), namely Scenario 1 (maximum MT lengths and MT-to-MT lateral access), Scenario 2 (maximum MT lengths with no lateral movement permitted), Scenario 4 (25% proximal staggering), and Scenario 7 (25% distal staggering). For each scenario and influx rate, four dynamicity maps are shown that represent data for multiple conditions that are Kin3 (F) and Kin1 (S) separately, both motors combined, and Kin1 (S) alone on an expanded x-axis scale. In Scenario 1, which is least restrictive for MT access, the rate of detachment for both Kin1 (S) and Kin3 (F) is nominal at 10 motors/s (Figure 5A, central column, note scales, and dispersed peaks). The Kin3 (F) processivity is equivalent to the MT maximum length in our defined parameters, and the allowance of MT-to-MT transitions results in minimal dissociations affected by Kin1 (S) in Scenario 1 at 10 motors/s. At an increased 15 motor/s, the dynamicity of both Kin1 (S) and Kin3 (F) increases due to a higher crowding of motors in between MT lanes (Figure 5A, right, note scales, and compacting peaks). In Scenario 2, when no lateral MT-to-MT access is permitted (Figure 5B), the flow rate and composition of bound motors on MTs are dictated by slower Kin1 (S). This is due to the forced dissociation of Kin3 (F) when it trails and encounters Kin1 (S) (Figure 5B central column, note scales, and elevated compacted dynamicity plot). The elevated blue peaks are in accordance with the high dynamic on-off characteristic of Kin3 (F) along the bulk. In scenarios with proximal (Scenario 4, Figure 5C) or distal staggering (Scenario 7, Figure 5D) and a slower flow rate of 10 motors/s, the Kin1 (S) motor is nominally affected, whereas Kin3 (F) due to hindrance along the singular MT lane by the slower Kin3 (S) has increased dissociation to the productive reservoir and dynamic on–off flow in channeled regions. At 15 motors/s, both kinesins are limited in progression, with increased dissociation and limited ability to reassociate due to crowded conditions, effectively gating the downstream flow of motors and the type of motors progressing (Figure 5C, central and right columns, note scales). In situations involving distal staggering and high influx rates of 15 motors/s (Figure 5D, right, note scale), we observed bulk detachment of both motors, resulting in a much crowded and channelized flow with limited progressive movement due to limited access to rejoin MTs. This is reflected in a flatter dynamic plot, indicating a narrow band of flow. The distribution and flow dynamics of the kinesins along an MMLS and productive reservoir in two staggered scenarios (Scenarios 4 and 7) are shown in Supplementary Videos 1, 2. Overall, our findings suggest that under high flow rates of 15 motors/s, the channelization of motors at the distal end of the MTs affects the dissociation of both faster and slower motors. The results of our DyNAMO simulations highlight that the alignment of axonal MT organization with different kinesin parameters, such as speed and processivity, plays an important role in the spatial flow of kinesin types and concentrations along the axonal MT network.

### 3.5. DyNAMO analysis of multiple injury scenarios along the axon length and signal outputs

**Figure 6:**
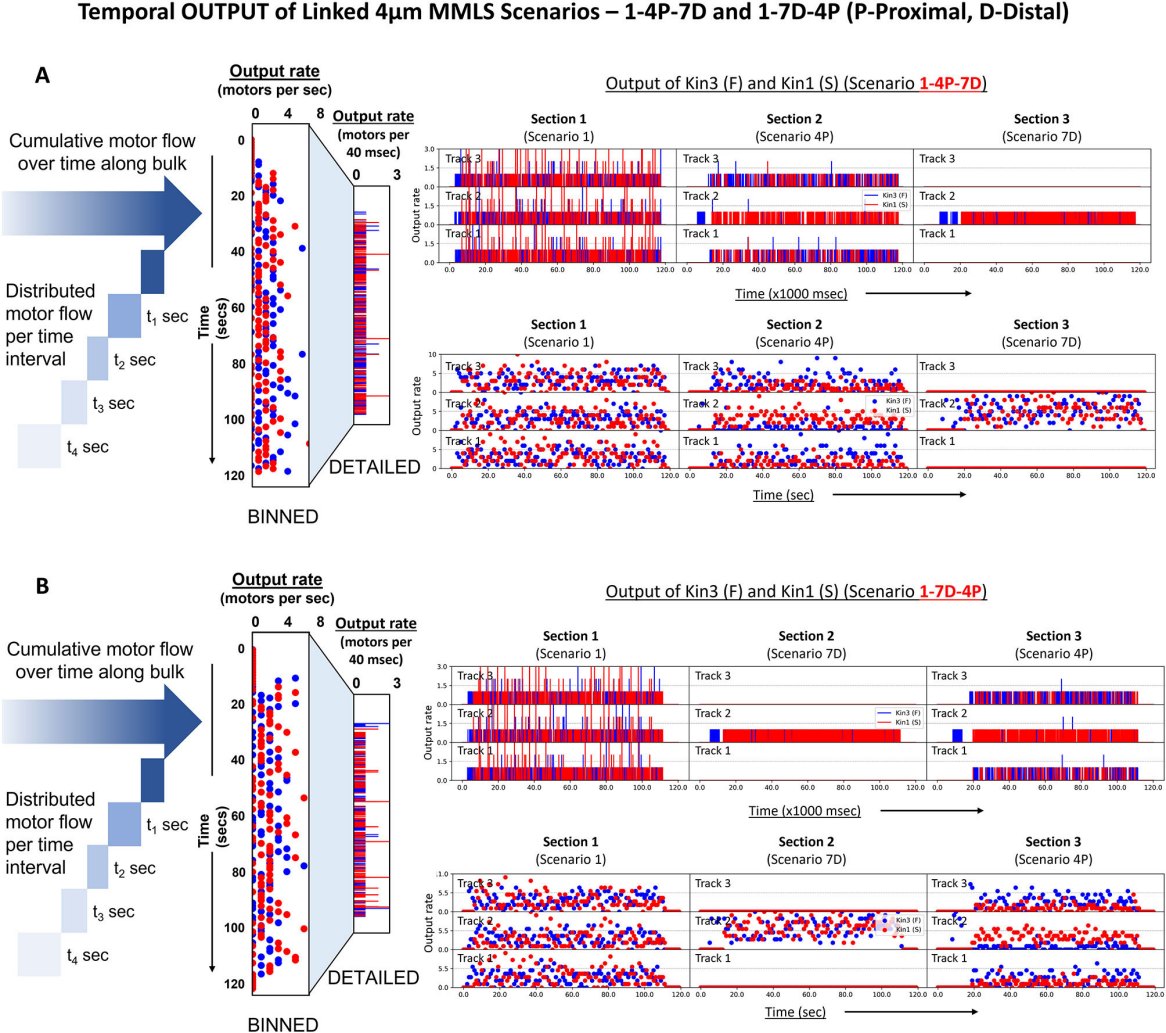
Temporal motor output from combined normal and injury-linked MMLS scenarios. Different scenarios and their restrictions dictate the final temporal output of kinesins along the axon length. The figure shows the temporal outflow of motors from different lanes of a combined multi-MMLS. Maximum MT length of 4 μm. In **(A, B)** the left figure illustrates motor delivery either as “DETAILED” per 40 ms time step or “BINNED” within a 1-s timeframe, equivalent to 25 iterations. [**(A)**—right] The rotated scatter plots show a varied range of dynamicity of motors for different scenarios. In multi-MMLS Scenario 1-4P-7D, we see that the outflow of motors is equally distributed along the three parallel MT lanes of Section 1 (Scenario 1), consistent with the provision of lateral movement. However, for Section 2 (Scenario 4P), proximal staggering channelizes motors at entry. Beyond the staggering point due to overcrowding of motors, most of the slower motors (red) remain in the middle lane forcing the faster motors (blue) to move to adjacent lanes (here Lanes 1 and 3) as evident from the increased blue dots. In Section 3 (Scenario 7D), all motors are channelized to a singular middle lane at the opposite distal end, which results in a compressed output. [**(B)**—right] Similar dynamics can be seen for sections in Scenario 1-7D-4P.

To gain a better understanding of how motors flow in bulk along the axon in different scenarios and segments, we developed a multi-MMLS heuristic consisting of three linked MMLS scenarios and a junctional reservoir. We computed the outflow rate of each motor from each individual lane/MT of the MMLS to assess the impact of different scenarios on the output flow of Kin3 (F) and Kin1 (S). We show two methods for representing motor delivery at different rates: “DETAILED” flow, which shows motor delivery at every computation timestep (40 ms; Figures 6A, B, upper rows), and “BINNED” data, which is a collective output over a timeframe of 1 s, equivalent to 25 iterations (Figures 6A, B, bottom rows). To illustrate the temporal output dynamics, we also generated simulation videos (Supplementary Videos 3, 4) for specific MMLS Scenarios 4 and 7. The temporal output of the two motor types was tracked through each of the single scenario MT sections of the multi-MMLS scenarios from left to right. Each dot along the time (x) axis represents the cumulative number of motors output from each MT section per second (values indicated on the y-axis; Figures 6A, B, right). In the multi-MMLS Scenario 1-4P-7D (P-25% proximal staggering and D-25% distal staggering), the binned plot in Section 1 reveals that motors are equally output from all three lanes due to the provision of lateral movement on adjacent lanes that avoids crowding. As motors enter and exit Section 2, proximal staggering exists, and the slower motor Kin1 (S) dominates the singular channeled lane (Lane 2), while faster motors tend to move to adjacent lanes (Lanes 1 and 3). The output of Section 2 reflects the pattern established by the proximal staggering, as motors retain their favorable positions. Entering and exiting Section 3, since all the outflows are channelized at the distal end to a single lane (Lane 2), the output reflects a restored mixed motor composition. When a different MMLS Scenario 1-7D-4P is evaluated in which distal and proximal staggering are adjacent, the final output from each MT is reflective of the previous injury event. In both evaluated multi-MMLS scenarios, we observe that the temporal outflow rate of kinesins is ~4–5 motors/s for all sections. To further investigate the relationship of kinesin processivity and MT length with the temporal outflow, we simulated similar multi-MMLS scenarios with a longer MT length of 8 μm (Supplementary Figure 1). We observe that for longer MT lengths within the multi-MMLS, the temporal outflow rate is comparatively lower (~2–3 motors/s), an indication that MT displacements of kinesins, on/off dynamicity, and loss of kinesins from the productive reservoir are greater.

**Figure 7:**
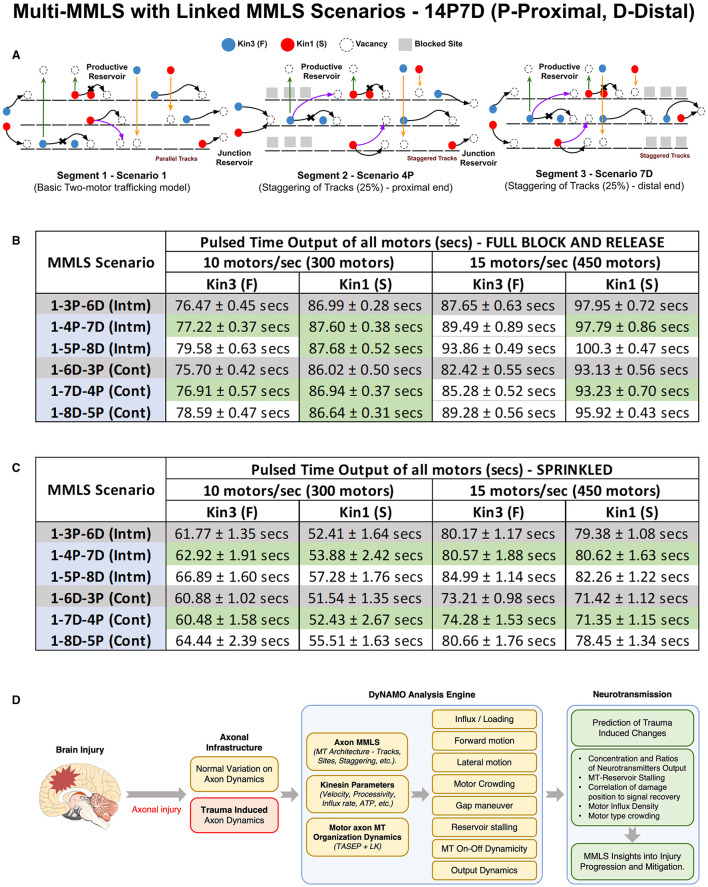
Intermittent axonal injuries are more disruptive than single broader impacts. Illustrated and quantified is the comprehensive flow of the dual kinesins along the axon length when encountering differing injury scenarios in a multi-MMLS format evaluated at influx rates of 10 and 15 motors/s. **(A)** Representative diagram of the multi-MMLS Scenario 1-4P-7D. The other multi-MMLS scenarios used in **(B, C)** are not shown. In **(B, C)** we provide an input pulse of kinesins for 30 s and then measure the time required for all motors to exit the system. We include benchmark normal multi-MMLS scenarios (1-3P-6D and 1-6D-3P), at 10 or 15 motors/s influx. The data output is shown for spaced/intermediate [intm] and clustered/continuous [cont] axonal injuries. In **(B)** we use a full block and release multi-MMLS scenario, such that the Kin3 (F) and Kin1 (S) influx occurs on an empty MT MMLS from the minus end to delivery to MT plus ends. In **(C)** we model scenarios assuming an initial semi-dense “sprinkled” distribution of motors on the multi-MMLS and new motors entering that MT injury region. For **(B, C)** our [intm] TBI MMLS scenarios shows a significant delay vs. normal controls [gray] in the throughput of both the Kin1 (S) and Kin3 (F). When an initial semi-dense “sprinkled” state of the multi-MMLS scenarios is used the effect of crowding is consistent across the scenarios but with substantial delay to output. In comparing [intm] Scenarios (1-4P-7D and 1-5P-8D), there is a reduced and gated flow of downstream motors for Kin1 (S) and Kin3 (F) compared to the similar influx conditions for [cont] axonal injury (1-7D-4P). **(D)** The DyNAMO analysis engine is a parameter-dense platform that provides new information to understand underlying changes in axonal transport in the context of axonal injury to benefit downstream diagnostics and therapies for traumatic brain injury and other axonopathies.

In Figure 6, we evaluated the motor distribution output for different multi-MMLS scenarios under a continuous motor flow rate of 10 motors/s. We next evaluated the impact of a pulsed input of motors in several multi-MMLS scenarios at 10/15 motors/s. We either generate a “fresh” block and release it to free MTs (Figure 7B) or we assume MTs already contain residual motors “sprinkled” due to “previous” unblocked trafficking in that region (Figure 7C). The modeled distributed P-D multi-MMLS scenarios are 1-3P-6D (12.5%), 1-4P-7D (25%) (Figure 7A), and 1-5P-8D (50%), and D-P multi-MMLS scenarios that create a larger injury site are 1-6D-3P (12.5% staggering), 1-7D-4P (25% staggering), and 1-8D-6P (50% staggering). All injury scenarios (25 or 50% staggering) are compared to the 12.5% staggering considered within the normal range (Figures 7B, C, gray rows). We applied a pulsed input for 15 s which involved 300–450 motors for both Kin1 (S) and Kin3 (F) at association rates of 10 and 15 motors per second, respectively. We recorded the time it took for all the motors to exit Section 3 (pulsed time output) in each multi-MMLS scenario, and the results are listed in the tables (Figures 7B, C). The green-shaded ranges are near-normal (gray shading). When intermittent successive regions of staggering are present, such as in multi-MMLS P-D scenarios, kinesins take longer to exit (delayed progression) than when a larger single continuous staggered region (our multi-MMLS D-P scenarios) is present. Thus, DyNAMO predicts that smaller intermittent damage is more disruptive in injury scenarios. The higher flow rate in injury scenarios coupled with pulsed input in a sprinkled multi-MMLS, generates the greatest delays in output for both motors. Kin3 (F) is affected by “full” block and release for 15 motors/s, while Kin3 (F) and Kin1 (S) are also affected by slower inflow in MMLS for a higher percentage of staggering. All these data are consistent with counteracting factors of motor speed, processivity, MT organization, and crowding.

## 4. Discussion

Mathematical models of neuronal axons complement and expand biological studies by providing a missing correlation between nanoscale mechanisms and spatio-temporal dynamic events relevant to neuronal function and signaling. The nanoscale dynamics of motors along the axon's dense polar (Burton and Paige, 1981) naturally staggered and discontinuous MT networks (Baas et al., 2016; Prokop, 2020) in living biological systems are still largely undescribed experimentally. This limits the full understanding of the normal and perturbed dynamic mechanisms of multiple interacting biomolecules in living systems. Moreover, surprisingly few studies exist that have detailed regional maps of axonal MT organization (Chalfie and Thomson, 1979; Yu and Baas, 1994; Reis et al., 2012), and virtually no study exists that map axonal MT organization and staggering distribution along the entire length. Due to limited nanoscale experimental correlations that reflect dynamics, axonopathies and axonal transport deficits in neurological disorders remain largely mysterious, hindering our ability to unravel neuropathology mechanisms (Millecamps and Julien, 2013). The DyNAMO theoretical model of axonal transport was developed to reveal the complex interrelationship between normal and injury-perturbed MT axonal architecture and the impacts on interactions of multiple motor protein types traversing that infrastructure. DyNAMO permits multi-parameter co-evaluations relevant to axonal injury (Figure 7D), including multiple kinesin types with distinct speed and processivity motility parameters, the ability to influence influx rate, and to track motor movements as MT-to-MT, detachment/reassociation dynamicity, stationary states, or forward progression, and in the context of axonal MT injuries reflected in MT length (or blocked access) and MT staggering that are regional or distributed lengthwise. No other model currently exists with this complexity to simulate axonal transport.

DyNAMO provides modeling of axonal injury and is expandable to consider broader axonal mechanisms and applications. In the context of TBI and CTE, DyNAMO is expected to help identify, classify, and model disease hallmarks and biomarkers relevant to injury severity by kinesins and their cargos of vesicles, organelles, RNA, proteins, and neurotransmitter signaling molecules, For example, recent studies indicate that TDP-43, an indicator of severe damage to axons, is transported by KHC in neurons, Kin1 (S) modeled here, with the overexpression or inhibition of KHC leading to an increase or decrease, respectively, in the transport of TDP-43 (Chu et al., 2019). DyNAMO analysis is consistent with this finding and reveals an impressive tolerance of slower Kin1 (S) kinesin to minor alterations in MT infrastructure, with effects only seen with more severe damage scenarios, supporting TDP-43 as a severe injury hallmark. In contrast, we observe immediate effects with minor damage on faster kinesins such as Kin3 (F) and their cargos. In relevance to signaling output, DyNAMO reveals how axonal injuries can delay signaling and generate altered signaling patterns, composition, and strengths in relevance to kinesin types. DyNAMO allows modeling multiple kinesins in relation to perturbations to axon MT infrastructure in this study, relevant to injury states (Matamoros and Baas, 2016; Muñoz-Lasso et al., 2020) but is generalizable to address a multitude of future questions on additional mechanisms that impact kinesin access to MTs in the context of MT-associated proteins (MAPs), such as CAMSAP (Jiang et al., 2014; Yau et al., 2014) and MAPT (Barbier et al., 2019), or post-translational tubulin modifications (Janke and Chloë Bulinski, 2012) postulated to represent a code for binding interactions, as well as MT length regulating agents (Conze et al., 2022). We also demonstrate DyNAMO's flexibility to evaluate additional parameters, such as reduced local levels of ATP available to kinesins in injury zones, a consequence of disrupted mitochondrial transport in neurodegeneration (Sleigh et al., 2019; Supplementary Figure 2). By subclassifying our position-specific productive reservoir, we regionally evaluated ATP concentration differences relevant to injury and non-injury sites. In the case of low ATP concentration, we defined a longer time constraint on kinesins vs. no time restriction on normal ATP levels in non-injury regions. The simulation output reveals that when the availability of ATP is reduced, there is delayed output flow of both motor types, with a higher impact on Kin1 (S) low processivity kinesin, and with output further influenced by the damage scenarios. In DyNAMO, any impact that removes a motor from the MT to the productive reservoir (i.e., crowding or limit of processivity or lack of ATP) offers an opportunity to control motor stationary position vs. forward motion, or in future studies, a switch between the anterograde and retrograde movement of cargo. Although in this study we focus on kinesin anterograde transport and MT axonal injury scenarios, intracellular transport of cargo is often bidirectional. This is due in part to retaining a mixed steady-state population of opposite polarity unidirectional kinesin and dynein motors (Encalada et al., 2011). Simulations will help distinguish multiple mechanisms that may include the ratio of motor types in a tug of war (Gross, 2004; Welte, 2004) or regulation of motor activity, including autoinhibition (Verhey and Hammond, 2009; Akhmanova and Hammer, 2010) or KLC interactions between the intact Kinesin-1C/KLC1 complex and dynein, which were shown to be needed for proper dynein retrograde activity (Martin et al., 1999; Ally et al., 2009; Encalada et al., 2011). Since a switch to the retrograde motion of motor-coated vesicles may impact several local levels of kinesins, consideration of the influx of the new kinesins will also need to be evaluated. In our studies with DyNAMO, we note that the rate of influx of motors has interestingly different impacts on kinesin types. We also anticipate the use of DyNAMO in the analysis of additional neuronal kinesins (Hirokawa and Takemura, 2005; Hirokawa et al., 2009) or evaluating the impact of multiple kinesins on processivity and obstacle avoidance (Ferro et al., 2019). DyNAMO reveals how axonal MT organization and multiple kinesins interplay to generate localized axonal crowding and patterning. Actin rings have also been proposed to play a role in localized crowding along the axon length (Sood et al., 2018). To what degree these two mechanisms, axonal MT staggering and actin rings, may co-contribute to the axonal flow of information is not yet known. In summary, DyNAMO is a powerful expandable platform for rigorous investigation of axonal transport mechanisms that reflect biological mechanisms and disease.

Finally, we anticipate that DyNAMO and other sub-neuronal models will also benefit the composition of larger neuronal communication models. We previously modeled the neuron as a nanoscale communication system using experimental data from multiple biological studies to understand and compare amyloid-beta toxicity and altered calcium signaling in Alzheimer's disease (Banerjee et al., 2018) in end-to-end events in intracellular signaling. It is anticipated that neuronal compartment models such as DyNAMO for axonopathies will advance biomedical research, analogous to models of synapses to evaluate synaptopathies for autism spectrum disorders (Chatterjee et al., 2021) and the spatial dendritic context of synaptic signaling (Larkum and Nevian, 2008; Tønnesen and Nägerl, 2016) in cognition and psychiatry. New initiatives toward mapping the spatial three-dimensional cellular context of rodents and eventually the human brain also make it imperative to continue to build and strengthen models of neuronal function (McMenamin et al., 2003; Watanabe et al., 2004; Pa?ca, 2018). Fully developed software and hardware computational models that interlink synaptic function, dendritic mechanisms, somatic correlations, and axonal signaling are almost certain to be as complex as larger topological models of brain function (Hennig, 2013; Chen and Sneyd, 2015). DyNAMO represents an innovative significant step in deciphering, visualizing, and quantifying axonal MT cytoskeleton end-to-end transport mechanisms in order to keep pace with the experimental realization of nanoscale structures and processes in biological systems. DyNAMO use will benefit studies relevant to a range of axonopathy diseases and mechanisms, including relevance to brain trauma or cognitive disorders such as Alzheimer's disease. The greatest challenge in modeling biological aspects of axonal transport with DyNAMO is expected to primarily be in computing power; currently, simulations are run on a laptop or small GPU cluster, whereas more complex parameters and interrelationships are expected to expand this need, particularly for full three-dimensional complex interrelationships. Where experimental detail is lacking, assumptions must be generated that may have compounding downstream effects. Because our focus was on the interrelationship of MT infrastructure and kinesin types, layers of kinesin regulation were not yet modeled in DyNAMO, such as multimers of kinesins that can affect processivity and bypassing obstacles, as well as dimerization that affects processivity. DyNAMO establishes a robust platform that can be built on by the user to make parameter and simulation decisions based on mechanistic questions.

## Data availability statement

The original contributions presented in the study are included in the article/Supplementary material. The DyNAMO algorithms can be found in online repositories at this link: https://github.com/soumyadeepchandra/DyNAMO.

## Author contributions

JP and AM conceived the project's experimental design. SC was primarily responsible for software algorithms and scenario data analysis. SC, ZO, and RC composed figures. JP, AM, and SC wrote most of the manuscript with contributions from all authors. All authors provided input to the mathematical framework, figures, and tables. All authors contributed to the article and approved the submitted version.

## References

[B1] Ahmadzadeh A. Noel A. Schober R. (2015). Analysis and design of multi-hop diffusion-based molecular communication networks. IEEE Trans. Mol. Biol. Multiscale Commun. 1, 144–157. 10.1109/TMBMC.2015.2501741

[B2] Ahmadzadeh H. Smith D. H. Shenoy V. B. (2014). Viscoelasticity of tau proteins leads to strain rate-dependent breaking of microtubules during axonal stretch injury: predictions from a mathematical model. Biophys. J. 106, 1123–1133. 10.1016/j.bpj.2014.01.024 24606936 PMC4026781

[B3] Akhmanova A. Hammer J. A. (2010). Linking molecular motors to membrane cargo. Curr. Opin. Cell Biol. 22, 479–487. 10.1016/j.ceb.2010.04.008 20466533 PMC3393125

[B4] Allen R. D. Metuzals J. Tasaki I. Brady S. T. Gilbert S. P. (1982). Fast axonal transport in squid giant axon. Science 218, 1127–1129. 10.1126/science.6183744 6183744

[B5] Ally S. Larson A. G. Barlan K. Rice S. E. Gelfand V. I. (2009). Opposite-polarity motors activate one another to trigger cargo transport in live cells. J. Cell Biol. 187, 1071–1082. 10.1083/jcb.200908075 20038680 PMC2806283

[B6] Baas P. W. Rao A. N. Matamoros A. J. Leo L. (2016). Stability properties of neuronal microtubules. Cytoskeleton 73, 442–460. 10.1002/cm.21286 26887570 PMC5541393

[B7] Baldwin K. R. Godena V. K. Hewitt V. L. Whitworth A. J. (2016). Axonal transport defects are a common phenotype in *Drosophila* models of ALS. Hum. Mol. Genet. 25, 2378–2392. 10.1093/hmg/ddw105 27056981 PMC5181624

[B8] Bálint Š. Verdeny Vilanova I. Sandoval Álvarez Á. Lakadamyali M. (2013). Correlative live-cell and superresolution microscopy reveals cargo transport dynamics at microtubule intersections. Proc. Natl. Acad. Sci. U. S. A. 110, 3375–3380. 10.1073/pnas.1219206110 23401534 PMC3587250

[B9] Banerjee A. Paluh J. Mukherjee A. Kumar K. G. Ghosh A. Naskar M. K. (2018). Modeling the neuron as a nanocommunication system to identify spatiotemporal molecular events in neurodegenerative disease. Int. J. Nanomed. 13, 3105–3128. 10.2147/IJN.S152664 29872297 PMC5975603

[B10] Barbier P. Zejneli O. Martinho M. Lasorsa A. Belle V. Smet-Nocca C.. (2019). Role of tau as a microtubule-associated protein: structural and functional aspects. Front. Aging Neurosci. 11, 204–204. 10.3389/fnagi.2019.00204 31447664 PMC6692637

[B11] Brunner C. Ernst K.-H. Hess H. Vogel V. (2004). Lifetime of biomolecules in polymer-based hybrid nanodevices. Nanotechnology 15, S540–S548. 10.1088/0957-4484/15/10/008

[B12] Burton P. R. Paige J. L. (1981). Polarity of axoplasmic microtubules in the olfactory nerve of the frog. Proc. Natl. Acad. Sci. U. S. A. 78, 3269–3273. 10.1073/pnas.78.5.3269 6973153 PMC319543

[B13] Chalfie M. Thomson J. N. (1979). Organization of neuronal microtubules in the nematode *Caenorhabditis elegans*. J. Cell Biol. 82, 278–289. 10.1083/jcb.82.1.278 479300 PMC2110421

[B14] Chatterjee R. Paluh J. L. Chowdhury S. Mondal S. Raha A. Mukherjee A. (2021). SyNC, a computationally extensive and realistic neural net to identify relative impacts of synaptopathy mechanisms on glutamatergic neurons and their networks in autism and complex neurological disorders. Front. Cell Neurosci. 15, 674030. 10.3389/fncel.2021.674030 34354570 PMC8330424

[B15] Che D. L. Chowdary P. D. Cui B. (2016). A close look at axonal transport: cargos slow down when crossing stationary organelles. Neurosci. Lett. 610, 110–116. 10.1016/j.neulet.2015.10.066 26528790 PMC4695265

[B16] Chen X. Sneyd J. (2015). A computational model of the dendron of the GnRH neuron. Bull. Math. Biol. 77, 904–926. 10.1007/s11538-014-0052-6 25503424

[B17] Chu J. F. Majumder P. Chatterjee B. Huang S. L. Shen C. K. J. (2019). TDP-43 regulates coupled dendritic mRNA transport-translation processes in co-operation with FMRP and Staufen1. Cell Rep. 29, 3118–3133.e6. 10.1016/j.celrep.2019.10.061 31801077

[B18] Ciandrini L. Neri I. Walter J. C. Dauloudet O. Parmeggiani A. (2014). Motor protein traffic regulation by supply–demand balance of resources. Phys. Biol. 11, e056006. 10.1088/1478-3975/11/5/056006 25204752

[B19] Conde C. Cáceres A. (2009). Microtubule assembly, organization and dynamics in axons and dendrites. Nat. Rev. Neurosci. 10, 319–332. 10.1038/nrn2631 19377501

[B20] Conze C. Trushina N. I. Holtmannspötter M. Rierola M. Attanasio S. Bakota L.. (2022). Super-resolution imaging and quantitative analysis of microtubule arrays in model neurons show that epothilone D increases the density but decreases the length and straightness of microtubules in axon-like processes. Brain Res. Bull. 190, 234–243. 10.1016/j.brainresbull.2022.10.008 36244582 PMC9634454

[B21] Cook L. J. Zia R. K. P. Schmittmann B. (2009). Competition between multiple totally asymmetric simple exclusion processes for a finite pool of resources. Phys. Rev. E Stat. Nonlin. Soft. Matter Phys. 80, 1–12. 10.1103/PhysRevE.80.031142 19905097

[B22] Coy D. L. Wagenbach M. Howard J. (1999). Kinesin takes one 8-nm step for each ATP that it hydrolyzes. J. Biol. Chem. 274, 3667–3671. 10.1074/jbc.274.6.3667 9920916

[B23] Daneshvar D. H. Goldstein L. E. Kiernan P. T. Stein T. D. McKee A. C. (2015). Post-traumatic neurodegeneration and chronic traumatic encephalopathy. Mol. Cell. Neurosci. 66, 81–90. 10.1016/j.mcn.2015.03.007 25758552

[B24] Derrida B. (1998). An exactly soluble non-equilibrium system: the asymmetric simple exclusion process. Phys. Rep. 301, 65–83. 10.1016/S0370-1573(98)00006-4

[B25] Derrida B. Evans M. R. (2009). “The asymmetric exclusion model: exact results through a matrix approach,” in Nonequilibrium Statistical Mechanics in One Dimension, ed V. Privman (Cambridge: Cambridge University Press), 277–304. 10.1017/CBO9780511564284.020

[B26] Dhiman I. Gupta A. K. (2016). Collective dynamics of an inhomogeneous two-channel exclusion process: theory and Monte Carlo simulations. J. Comput. Phys. 309, 227–240. 10.1016/j.jcp.2016.01.010

[B27] Dixit R. Ross J. L. Goldman Y. E. Holzbaur E. L. F. (2008). Differential regulation of dynein and kinesin motor proteins by tau. Science 319, 1086–1089. 10.1126/science.1152993 18202255 PMC2866193

[B28] Encalada S. E. Szpankowski L. Xia C. Goldstein L. S. B. (2011). Stable kinesin and dynein assemblies drive the axonal transport of mammalian prion protein vesicles. Cell 144, 551–565. 10.1016/j.cell.2011.01.021 21335237 PMC3576050

[B29] Feng Q. Mickolajczyk K. J. Chen G.-Y. Hancock W. O. (2018). Motor reattachment kinetics play a dominant role in multimotor-driven cargo transport. Biophys. J. 114, 400–409. 10.1016/j.bpj.2017.11.016 29401437 PMC5985011

[B30] Ferro L. S. Can S. Turner M. A. ElShenawy M. M. Yildiz A. (2019). Kinesin and dynein use distinct mechanisms to bypass obstacles. Elife 8, e48629. 10.7554/eLife.48629.028 31498080 PMC6783262

[B31] Fogarty C. E. Diwanji N. Lindblad J. L. Tare M. Amcheslavsky A. Makhijani K.. (2016). Extracellular reactive oxygen species drive apoptosis-induced proliferation via drosophila macrophages. Curr. Biol. 26, 575–584. 10.1016/j.cub.2015.12.064 26898463 PMC4765900

[B32] Ganguly A. Tang Y. Wang L. Ladt K. Loi J. Dargent B.. (2015). A dynamic formin-dependent deep F-actin network in axons. J. Cell Biol. 210, 401–417. 10.1083/jcb.201506110 26216902 PMC4523607

[B33] Gross S. P. (2004). Hither and yon: a review of bi-directional microtubule-based transport. Phys. Biol. 1, R1–R11. 10.1088/1478-3967/1/2/R01 16204815

[B34] Gupta A. K. (2016). Collective dynamics on a two-lane asymmetrically coupled TASEP with mutually interactive langmuir kinetics. J. Stat. Phys. 162, 1571–1586. 10.1007/s10955-016-1463-6

[B35] Hahn I. Voelzmann A. Liew Y. T. Costa-Gomes B. Prokop A. (2019). The model of local axon homeostasis—Explaining the role and regulation of microtubule bundles in axon maintenance and pathology. Neural Dev. 14, 11. 10.1186/s13064-019-0134-0 31706327 PMC6842214

[B36] Handley E. E. Pitman K. A. Dawkins E. Young K. M. Clark R. M. Jiang T. C.. (2016). Synapse dysfunction of layer V pyramidal neurons precedes neurodegeneration in a mouse model of TDP-43 proteinopathies. Cerebr. Cortex 27, 3630–3647. 10.1093/cercor/bhw185 27496536

[B37] Hennig M. H. (2013). Theoretical models of synaptic short term plasticity. Front. Comput. Neurosci. 7, 1–10. 10.3389/fncom.2013.00154 23626536 PMC3630333

[B38] Hirokawa N. Noda Y. Tanaka Y. Niwa S. (2009). Kinesin superfamily motor proteins and intracellular transport. Nat. Rev. Mol. Cell Biol. 10, 682–696. 10.1038/nrm2774 19773780

[B39] Hirokawa N. Takemura R. (2005). Molecular motors and mechanisms of directional transport in neurons. Nat. Rev. Neurosci. 6, 201–214. 10.1038/nrn1624 15711600

[B40] Hoeprich G. J. Thompson A. R. McVicker D. P. Hancock W. O. Berger C. L. (2014). Kinesin's neck-linker determines its ability to navigate obstacles on the microtubule surface. Biophys. J. 106, 1691–1700. 10.1016/j.bpj.2014.02.034 24739168 PMC4008791

[B41] Janke C. Chloë Bulinski J. (2012). Post-translational regulation of the microtubule cytoskeleton: mechanisms and functions. Nat. Rev. Mol. Cell Biol. 13, 276–276. 10.1038/nrm3310 22086369

[B42] Jenkins B. Decker H. Bentley M. Luisi J. Banker G. (2012). A novel split kinesin assay identifies motor proteins that interact with distinct vesicle populations. J. Cell Biol. 198, 749–761. 10.1083/jcb.201205070 22908316 PMC3514038

[B43] Jiang K. Hua S. Mohan R. Grigoriev I. Yau K. W. Liu Q.. (2014). Microtubule minus-end stabilization by polymerization-driven CAMSAP deposition. Dev. Cell 28, 295–309. 10.1016/j.devcel.2014.01.001 24486153

[B44] Jiang R. Hu M. Bin Wu Y. H. Wu Q. S. (2008). Weak and strong coupling in a two-lane asymmetric exclusion process. Phys. Rev. E Stat. Nonlin. Soft Matter. Phys. 77, e041128. 10.1103/PhysRevE.77.041128 18517599

[B45] Jiang R. Wang R. Wu Q.-S. (2007). Two-lane totally asymmetric exclusion processes with particle creation and annihilation. Physica A 375, 247–256. 10.1016/j.physa.2006.08.025

[B46] Kapitein L. C. Hoogenraad C. C. (2015). Building the neuronal microtubule cytoskeleton. Neuron 87, 492–506. 10.1016/j.neuron.2015.05.046 26247859

[B47] Kojima H. Muto E. Higuchi H. Yanagida T. (1997). Mechanics of single kinesin molecules measured by optical trapping nanometry. Biophys. J. 73, 2012–2022. 10.1016/S0006-3495(97)78231-6 9336196 PMC1181101

[B48] Larkum M. E. Nevian T. (2008). Synaptic clustering by dendritic signalling mechanisms. Curr. Opin. Neurobiol. 18, 321–331. 10.1016/j.conb.2008.08.013 18804167

[B49] Ledbetter M. C. Porter K. R. (1964). Morphology of microtubules of plant cell. Science 144, 872–874. 10.1126/science.144.3620.872 17733620

[B50] Leduc C. Padberg-Gehle K. Varga V. Helbing D. Diez S. Howard J. (2012). Molecular crowding creates traffic jams of kinesin motors on microtubules. Proc. Natl. Acad. Sci. U. S. A. 109, 6100–6105. 10.1073/pnas.1107281109 22431622 PMC3341076

[B51] Lessard D. V. Zinder O. J. Hotta T. Verhey K. J. Ohi R. Berger C. L. (2019). Polyglutamylation of tubulin's C-terminal tail controls pausing and motility of kinesin-3 family member KIF1A. J. Biol. Chem. 294, 6353–6363. 10.1074/jbc.RA118.005765 30770469 PMC6484136

[B52] Leterrier C. (2016). “The axon initial segment, 50 years later,” in Current Topics in Membranes (Amsterdam: Elsevier), 185–233. 10.1016/bs.ctm.2015.10.005 26781833

[B53] Leterrier C. (2021). A pictorial history of the neuronal cytoskeleton. J. Neurosci. 41, 11–27. 10.1523/JNEUROSCI.2872-20.2020 33408133 PMC7786211

[B54] Leterrier C. Dubey P. Roy S. (2017). The nano-architecture of the axonal cytoskeleton. Nat. Rev. Neurosci. 18,713–726. 10.1038/nrn.2017.129 29097785

[B55] Liang W. H. Li Q. Rifat Faysal K. M. King S. J. Gopinathan A. Xu J. (2016). Microtubule defects influence kinesin-based transport *in vitro*. Biophys. J. 110, 2229–2240. 10.1016/j.bpj.2016.04.029 27224488 PMC4880806

[B56] Ling S. C. (2018). Synaptic paths to neurodegeneration: the emerging role of TDP-43 and FUS in synaptic functions. Neural Plast 2018, 1–13. 10.1155/2018/8413496 29755516 PMC5925147

[B57] Lo K. Y. Kuzmin A. Unger S. M. Petersen J. D. Silverman M. A. (2011). KIF1A is the primary anterograde motor protein required for the axonal transport of dense-core vesicles in cultured hippocampal neurons. Neurosci. Lett. 491, 168–173. 10.1016/j.neulet.2011.01.018 21256924

[B58] Lu B. Vogel H. (2009). *Drosophila* models of neurodegenerative diseases. Ann. Rev. Pathol. 4, 315–342. 10.1146/annurev.pathol.3.121806.151529 18842101 PMC3045805

[B59] Martin M. Iyadurai S. J. Gassman A. Gindhart J. G. Hays T. S. Saxton W. M. (1999). Cytoplasmic dynein, the dynactin complex, and kinesin are interdependent and essential for fast axonal transport. Mol. Biol. Cell 10, 3717–3728. 10.1091/mbc.10.11.3717 10564267 PMC25669

[B60] Matamoros A. J. Baas P. W. (2016). Microtubules in health and degenerative disease of the nervous system. Brain Res. Bull. 126, 217–225. 10.1016/j.brainresbull.2016.06.016 27365230 PMC5079814

[B61] McKee A. C. Stein T. D. Nowinski C. J. Stern R. A. Daneshvar D. H. Alvarez V. E.. (2013). The spectrum of disease in chronic traumatic encephalopathy. Brain 136, 43–64. 10.1093/brain/aws307 23208308 PMC3624697

[B62] McMenamin P. G. Wealthall R. J. Deverall M. Cooper S. J. Griffin B. (2003). Macrophages and dendritic cells in the rat meninges and choroid plexus: three-dimensional localisation by environmental scanning electron microscopy and confocal microscopy. Cell Tissue Res. 313, 259–269. 10.1007/s00441-003-0779-0 12920643

[B63] Millecamps S. Julien J. P. (2013). Axonal transport deficits and neurodegenerative diseases. Nat. Rev. Neurosci. 14, 161–176. 10.1038/nrn3380 23361386

[B64] Muñoz-Lasso D. C. Romá-Mateo C. Pallardó F. V. Gonzalez-Cabo P. (2020). Much more than a scaffold: cytoskeletal proteins in neurological disorders. Cells 9, 358. 10.3390/cells9020358 32033020 PMC7072452

[B65] Nagano S. Jinno J. Abdelhamid R. F. Jin Y. Shibata M. Watanabe S.. (2020). TDP-43 transports ribosomal protein mRNA to regulate axonal local translation in neuronal axons. Acta Neuropathol. 140, 695–713. 10.1007/s00401-020-02205-y 32803350

[B66] Nishinari K. Okada Y. Schadschneider A. Chowdhury D. (2005). Intracellular transport of single-headed molecular motors KIF1A. Phys. Rev. Lett. 95, 118101. 10.1103/PhysRevLett.95.118101 16197050

[B67] Nixon R. A. Shea T. B. (1992). Dynamics of neuronal intermediate filaments: a developmental perspective. Cell Motil. Cytoskelet. 22, 81–91. 10.1002/cm.970220202 1633625

[B68] Okada Y. Higuchi H. Hirokawa N. (2003). Processivity of the single-headed kinesin KIF1A through biased binding to tubulin. Nature 424, 574–577. 10.1038/nature01804 12891363

[B69] Pa?ca S. P. (2018). The rise of three-dimensional human brain cultures. Nature 553, 437–445. 10.1038/nature25032 29364288

[B70] Papandréou M. J. Leterrier C. (2018). The functional architecture of axonal actin. Mol. Cell. Neurosci. 91, 151–159. 10.1016/j.mcn.2018.05.003 29758267

[B71] Parmeggiani A. Franosch T. Frey E. (2003). Phase coexistence in driven one-dimensional transport. Phys. Rev. Lett. 90, e086601. 10.1103/PhysRevLett.90.086601 12633448

[B72] Parmeggiani A. Franosch T. Frey E. (2004). Totally asymmetric simple exclusion process with Langmuir kinetics. Phys. Rev. E 70, e046101. 10.1103/PhysRevE.70.046101 15600454

[B73] Petersen J. D. Kaech S. Banker G. (2014). Selective microtubule-based transport of dendritic membrane proteins arises in concert with axon specification. J. Neurosci. 34, 4135–4147. 10.1523/JNEUROSCI.3779-13.2014 24647935 PMC3960460

[B74] Prokop A. (2020). Cytoskeletal organization of axons in vertebrates and invertebrates. J. Cell Biol. 219, e201912081. 10.1083/jcb.201912081 32369543 PMC7337489

[B75] Pronina E. Kolomeisky A. B. (2004). Two-channel totally asymmetric simple exclusion processes. J. Phys. A Math. Gen. 37, 9907–9918. 10.1088/0305-4470/37/42/005

[B76] Pronina E. Kolomeisky A. B. (2006). Asymmetric coupling in two-channel simple exclusion processes. Physica A 372, 12–21. 10.1016/j.physa.2006.05.006

[B77] Rank M. Frey E. (2018). Crowding and pausing strongly affect dynamics of kinesin-1 motors along microtubules. Biophys. J. 115, 1068–1081. 10.1016/j.bpj.2018.07.017 30146266 PMC6139881

[B78] Reis G. F. Yang G. Szpankowski L. Weaver C. Shah S. B. Robinson J. T.. (2012). Molecular motor function in axonal transport *in vivo* probed by genetic and computational analysis in *Drosophila*. Mol. Biol. Cell 23, 1700–1714. 10.1091/mbc.e11-11-0938 22398725 PMC3338437

[B79] Ruthel G. Banker G. (1999). Role of moving growth cone-like “wave” structures in the outgrowth of cultured hippocampal axons and dendrites. J. Neurobiol. 39, 97–106. 10.1002/(SICI)1097-4695(199904)39:1<97::AID-NEU8>3.0.CO;2-Z 10213456

[B80] Shigeoka T. Jung H. Jung J. Turner-Bridger B. Ohk J. Lin J. Q.. (2016). Dynamic axonal translation in developing and mature visual circuits. Cell 166, 181–192. 10.1016/j.cell.2016.05.029 27321671 PMC4930487

[B81] Sleigh J. N. Rossor A. M. Fellows A. D. Tosolini A. P. Schiavo G. (2019). Axonal transport and neurological disease. Nat. Rev. Neurol. 15, 691–703. 10.1038/s41582-019-0257-2 31558780

[B82] Smith R. S. (1980). The short term accumulation of axonally transported organelles in the region of localized lesions of single myelinated axons. J. Neurocytol. 9, 39–65. 10.1007/BF01205226 6162922

[B83] Song Y. Kang M. Morfini G. Brady S. T. (2016). Fast axonal transport in isolated axoplasm from the squid giant axon. Methods Cell Biol. 4, 331–348. 10.1016/bs.mcb.2015.07.004 26794522

[B84] Sood P. Murthy K. Kumar V. Nonet M. L. Menon G. I. Koushika S. P. (2018). Cargo crowding at actin-rich regions along axons causes local traffic jams. Traffic 19, 166–181. 10.1111/tra.12544 29178177 PMC13083050

[B85] Soppina V. Norris S. R. Dizaji A. S. Kortus M. Veatch S. Peckham M.. (2014). Dimerization of mammalian kinesin-3 motors results in superprocessive motion. Proc. Natl. Acad. Sci. U. S. A. 111, 5562–5567. 10.1073/pnas.1400759111 24706892 PMC3992690

[B86] Spohn H. (1991). Large Scale Dynamics of Interacting Particles. Berlin; Heidelberg: Springer Berlin Heidelberg.

[B87] Sui H. Downing K. H. (2010). Structural basis of interprotofilament interaction and lateral deformation of microtubules. Structure 18, 1022–1031. 10.1016/j.str.2010.05.010 20696402 PMC2976607

[B88] Sun F. Zhu C. Dixit R. Cavalli V. (2011). Sunday Driver/JIP3 binds kinesin heavy chain directly and enhances its motility. EMBO J. 30, 3416–3429. 10.1038/emboj.2011.229 21750526 PMC3160654

[B89] Surana S. Villarroel Campos D. Lazo O. M. Moretto E. Tosolini A. P. Rhymes E. R.. (2020). The evolution of the axonal transport toolkit. Traffic 21, 13–33. 10.1111/tra.12710 31670447

[B90] Svoboda K. Schmidt C. F. Schnapp B. J. Block S. M. (1993). Direct observation of kinesin stepping by optical trapping interferometry. Nature 365, 721–727. 10.1038/365721a0 8413650

[B91] Tilney L. G. Bryan J. Bush D. J. Fujiwara K. Mooseker M. S. Murphy D. B.. (1973). Microtubules: evidence for 13 protofilaments. J. Cell Biol. 59, 267–275. 10.1083/jcb.59.2.267 4805001 PMC2109099

[B92] Tønnesen J. Nägerl U. V. (2016). Dendritic spines as tunable regulators of synaptic signals. Front. Psychiatry 7, 101–101. 10.3389/fpsyt.2016.00101 27340393 PMC4899469

[B93] Trojanowski J. Walkenstein N. Lee V. (1986). Expression of neurofilament subunits in neurons of the central and peripheral nervous system: an immunohistochemical study with monoclonal antibodies. J. Neurosci. 6, 650–660. 10.1523/JNEUROSCI.06-03-00650.1986 2420946 PMC6568466

[B94] Tsukita S. Ishikawa H. (1980). The movement of membranous organelles in axons. Electron microscopic identification of anterogradely and retrogradely transported organelles. J. Cell Biol. 84, 513–530. 10.1083/jcb.84.3.513 6153657 PMC2110575

[B95] Vale R. Reese T. Sheetz M. (1985). Identification of a novel force-generating protein, kinesin, involved in microtubule-based motility. Cell 42, 39–50. 10.1016/S0092-8674(85)80099-4 3926325 PMC2851632

[B96] Vassilopoulos S. Gibaud S. Jimenez A. Caillol G. Leterrier C. (2019). Ultrastructure of the axonal periodic scaffold reveals a braid-like organization of actin rings. Nat. Commun. 10, 5803. 10.1038/s41467-019-13835-6 31862971 PMC6925202

[B97] Verbrugge S. van den Wildenberg S. M. J. L. Peterman E. J. G. (2009). Novel ways to determine kinesin-1′s run length and randomness using fluorescence microscopy. Biophys. J. 97, 2287–2294. 10.1016/j.bpj.2009.08.001 19843461 PMC2764061

[B98] Verhey K. J. Hammond J. W. (2009). Traffic control: regulation of kinesin motors. Nat. Rev. Mol. Cell Biol. 10, 765–777. 10.1038/nrm2782 19851335

[B99] Verma A. K. Gupta A. K. (2018). Limited resources in multi-lane stochastic transport system. J. Phys. Commun. 2, 045020. 10.1088/2399-6528/aabb3a

[B100] Verma A. K. Gupta A. K. Dhiman I. (2015). Phase diagrams of three-lane asymmetrically coupled exclusion process with langmuir kinetics. Europhys. Lett. 112, 30008. 10.1209/0295-5075/112/3000837198843

[B101] Vuijk H. D. Rens R. Vahabi M. MacKintosh F. C. Sharma A. (2015). Driven diffusive systems with mutually interactive langmuir kinetics. Phys. Rev. E 91, e032143. 10.1103/PhysRevE.91.032143 25871090

[B102] Wang R. Jiang R. Liu M. Liu J. Wu Q.-S. (2007). Effects of langmuir kinetics on two-lane totally asymmetric exclusion processes of molecular motor traffic. Int. J. Modern Phys. C 18, 1483–1496. 10.1142/S0129183107011479

[B103] Watanabe T. Frahm J. Michaelis T. (2004). Functional mapping of neural pathways in rodent brain *in vivo* using manganese-enhanced three-dimensional magnetic resonance imaging. NMR Biomed. 17, 554–568. 10.1002/nbm.937 15617054

[B104] Welte M. A. (2004). Bidirectional transport along microtubules. Curr. Biol. 14, R525–R537. 10.1016/j.cub.2004.06.045 15242636

[B105] Whitesides G. E. (1970). The art of computer programming. Nucl. Sci. Eng. 40, 358–358. 10.13182/NSE70-A19705

[B106] Xiao S. Cai J.-J. Liu F. Liu M. (2010). Theoretical investigation of synchronous totally asymmetric exclusion processes on lattices with a shortcut. Int. J. Mod. Phys. B 24, 5539–5546. 10.1142/S021797921005689X

[B107] Xu K. Zhong G. Zhuang X. (2013). Actin, spectrin, and associated proteins form a periodic cytoskeletal structure in axons. Science 339, 452–456. 10.1126/science.1232251 23239625 PMC3815867

[B108] Yamada K. M. Spooner B. S. Wessells N. K. (1971). Ultrastructure and function of growth cones and axons of cultured nerve cells. J. Cell Biol. 49, 614–635. 10.1083/jcb.49.3.614 4326456 PMC2108504

[B109] Yau K. W. van Beuningen S. F. B. Cunha-Ferreira I. Cloin B. M. C. van Battum E. Y. Will L.. (2014). Microtubule minus-end binding protein CAMSAP2 controls axon specification and dendrite development. Neuron 82, 1058–1073. 10.1016/j.neuron.2014.04.019 24908486

[B110] Yu W. Baas P. W. (1994). Changes in microtubule number and length during axon differentiation. J. Neurosci. 14, 2818–2829. 10.1523/JNEUROSCI.14-05-02818.1994 8182441 PMC6577472

