# Corrigendum: Axonal transport during injury on a theoretical axon

**DOI:** 10.3389/fncel.2023.1357885

**Published:** 2024-01-09

**Authors:** Soumyadeep Chandra, Rounak Chatterjee, Zachary T. Olmsted, Amitava Mukherjee, Janet L. Paluh

**Affiliations:** ^1^Electrical and Computer Science Engineering, Purdue University, West Lafayette, IN, United States; ^2^Department of Electronics, Electrical and Systems Engineering, University of Birmingham, Birmingham, United Kingdom; ^3^Nanobioscience, College of Nanoscale Science and Engineering, State University of New York Polytechnic Institute, Albany, NY, United States; ^4^Department of Neurosurgery, Ronald Reagan UCLA Medical Center, University of California, Los Angeles, Los Angeles, CA, United States; ^5^School of Computing, Amrita Vishwa Vidyapeetham (University), Kollam, Kerala, India

**Keywords:** traumatic brain injury, axonopathy, neurotransmission, microtubules, kinesins, TASEP-LK

In the published article, there was an error in the **Funding** statement. The **Funding** statement was incomplete. The correct **Funding** statement appears below.

“The Empire State Development's Division of Science, Technology, and Innovation (NYSTAR) Division funds 15 Centers for Advanced Technology (CATs) to encourage greater collaboration between private industry and universities in the development and application of new technologies. The CAT program, created in 1983, facilitates a continuing program of basic and applied research, development and technology transfer in multiple technological areas, in collaboration with and through the support of private industry. CATs play a critical role in spurring technology-based applied research and economic development in the state; promoting national and international research collaboration and innovation; and leveraging New York's research expertise and funding with investments from the federal government, foundations, businesses, venture capital firms and other entities. The work was supported by Center for Advanced Technology in Nanotechnology and Nanoelectronics funds to JP (CATN2 1186199-1-92476) as well as multi-university resources in a collaborative effort.”

The authors apologize for this error and state that this does not change the scientific conclusions of the article in any way. The original article has been updated.

