# Supplementary material for: Axonal transport during injury on a theoretical axon
Source: Front Cell Neurosci. 2023 Aug 11;17:1215945. doi: 10.3389/fncel.2023.1215945 (PMC10450981; doi:10.3389/fncel.2023.1215945)
Supplement: Supplementary file 5 [file Data_Sheet_1.pdf]

Supplementary Material

Axonal Transport During Injury on a Theoretical Axon

Soumyadeep Chandra[1], Rounak Chatterjee[2], Zachary T.Olmsted[3,4], Amitava Mukherjee[5,3], Janet L. Paluh[3*]

*** Correspondence:**
Janet L. Paluh,
paluhj@sunypoly.edu, jpaluh@albany.edu

1 Supplementary Data

Appendix 1: Implementation of TASEP-LK in context of complex mathematical parameters

When evaluating the behavior of multiple motors with different motility parameters, it is challenging to incorporate the complex interactions and dependencies between these motors and other dynamic variables into a unified set of solvable partial differential equations (PDEs). Parameters such as motor processivity and lifetime are interdependent and add additional complexity. To simplify the analysis, we use PDEs that remove these interdependencies and set specific testable conditions to examine these complex interactions. We combine multiple motility parameters, such as speed and processivity, into step-dependent equations to capture the individual motor variables in a combined system of multiple motors. However, incorporating processivity as a time-independent variable is difficult, as it is a state variable that changes with time and steps taken. We focused our analysis on two main actions that are lateral MT motor dynamics and stepping. The forward stepping of motors and their asymmetric coupling with adjacent MTs are governed by the Totally Asymmetric Simple Exclusion Process (TASEP) principle, but with an extended mobility of motors that can take multiple steps in one-time frame. The attachment and detachment of motors across productive reservoirs are governed by principles of Langmuir Kinetics (LK).

Let $\langle n_t^i \rangle$ and $\langle m_t^i \rangle$ denote the discrete binary state of occupancy of a lattice site (MT binding site) by particle kinesins A and B (kinesins examined) at site i in track t respectively. $\langle n_t^i \rangle = 1$ denotes site is occupied by kinesins A while $\langle n_t^i \rangle = 0$ denotes vacant site. Due to hard core repulsion $\langle n_t^i + m_t^i \rangle = 0 \; or \; 1$. For sites $1 \leq i \leq N-1$, the time evolution of $n_t^i \; and \; m_t^i$ are governed by the following equations on track 1:

1. $$\frac{d\langle n_1^i \rangle}{dt} = \omega_{a,R}\langle 1 - n_1^{i-1} n_1^i n_1^{i+1} - m_1^{i-1} m_1^i m_1^{i+1} \rangle - \omega_{a,D}\langle n_1^i \rangle + \langle n_1^{i-1}\left(1 - n_1^i - m_1^i\right)\rangle - \langle n_1^i\left(1 - n_1^{i+1} - m_1^{i+1}\right)\rangle - \omega_{a,l}\langle n_1^i n_1^{i+1}\left(1 - n_2^i - m_2^i\right)\left(1 - n_3^i - m_3^i\right)\rangle + \omega_{a,l}\langle n_2^i n_2^{i+1}\left(1 - n_1^i - m_1^i\right) + n_3^i n_3^{i+1}\left(1 - n_1^i - m_1^i\right)\rangle$$

2. $\frac{d\langle m_1^i\rangle}{dt} = \omega_{b,R}\langle 1 - n_1^{i-1}n_1^i n_1^{i+1} - m_1^{i-1}m_1^i m_1^{i+1}\rangle - \omega_{b,D}\langle m_1^i\rangle + \langle v m_1^{i-1}(1 - n_1^i - m_1^i)\rangle - \langle v m_1^i(1 - n_1^{i+1} - m_1^{i+1})\rangle - \omega_{b,l}\langle m_1^i m_1^{i+1}(1 - n_2^i - m_2^i)(1 - n_3^i - m_3^i)\rangle + \omega_{b,l}\langle m_2^i m_2^{i+1}(1 - n_1^i - m_1^i) + m_3^i m_3^{i+1}(1 - n_1^i - m_1^i)\rangle$

In stepwise motility, the forward hopping rate of kinesin 'a' is normalized to be a unit, and the forward hopping rate of 'b' is assumed to be $v \leq 1$. The hopping rate is usually given by biological properties. In the above equations 1 and 2 for kinesins 'a' and 'b', the positive and negative terms on the RHS denote the gain and loss terms arising due to reattachment and detachment to and from the productive reservoirs succeeded by forward hopping. The reattachment rate of kinesins 'a' and 'b' to any unoccupied lattice site i is denoted by $\omega_{a,R}$ *and* $\omega_{b,R}$, and the detachment rate from lattice site is denoted by $\omega_{a,D}$ *and* $\omega_{b,D}$. The movement of a motor to an adjacent track by lane changing is given by one loss term for track *t* to track *t-1, t+1* while two gain terms from track *t+1 or t-1* to track *t*. The lateral movement rate for Kinesins 'a' and 'b' is given by rate $\omega_{a,l}$ *and* $\omega_{b,l}$.

The corresponding equations for the other lanes are given as shown below:

3. $\frac{d\langle n_2^i\rangle}{dt} = \omega_{a,R}\langle 1 - n_2^{i-1}n_2^i n_2^{i+1} - m_2^{i-1}m_2^i m_2^{i+1}\rangle - \omega_{a,D}\langle n_2^i\rangle + \langle n_2^{i-1}(1 - n_2^i - m_2^i)\rangle - \langle n_2^i(1 - n_2^{i+1} - m_2^{i+1})\rangle - \omega_{a,l}\langle n_2^i n_2^{i+1}(1 - n_1^i - m_1^i)(1 - n_3^i - m_3^i)\rangle + \omega_{a,l}\langle n_1^i n_1^{i+1}(1 - n_2^i - m_2^i) + n_3^i n_3^{i+1}(1 - n_2^i - m_2^i)\rangle$

4. $\frac{d\langle m_2^i\rangle}{dt} = \omega_{b,R}\langle 1 - n_2^{i-1}n_2^i n_2^{i+1} - m_2^{i-1}m_2^i m_2^{i+1}\rangle - \omega_{b,D}\langle m_2^i\rangle + \langle v m_2^{i-1}(1 - n_2^i - m_2^i)\rangle - \langle v m_2^i(1 - n_2^{i+1} - m_2^{i+1})\rangle - \omega_{b,l}\langle m_2^i m_2^{i+1}(1 - n_1^i - m_1^i)(1 - n_3^i - m_3^i)\rangle + \omega_{b,l}\langle m_1^i m_1^{i+1}(1 - n_2^i - m_2^i) + m_3^i m_3^{i+1}(1 - n_2^i - m_2^i)\rangle$

5. $\frac{d\langle n_3^i\rangle}{dt} = \omega_{a,R}\langle 1 - n_3^{i-1}n_3^i n_3^{i+1} - m_3^{i-1}m_3^i m_3^{i+1}\rangle - \omega_{a,D}\langle n_3^i\rangle + \langle n_3^{i-1}(1 - n_3^i - m_3^i)\rangle - \langle n_3^i(1 - n_3^{i+1} - m_3^{i+1})\rangle - \omega_{a,l}\langle n_3^i n_3^{i+1}(1 - n_1^i - m_1^i)(1 - n_2^i - m_2^i)\rangle + \omega_{a,l}\langle n_1^i n_1^{i+1}(1 - n_3^i - m_3^i) + n_2^i n_2^{i+1}(1 - n_3^i - m_3^i)\rangle$

6. $\frac{d\langle m_3^i\rangle}{dt} = \omega_{b,R}\langle 1 - n_3^{i-1}n_3^i n_3^{i+1} - m_3^{i-1}m_3^i m_3^{i+1}\rangle - \omega_{b,D}\langle m_3^i\rangle + \langle v m_3^{i-1}(1 - n_3^i - m_3^i)\rangle - \langle v m_3^i(1 - n_3^{i+1} - m_3^{i+1})\rangle - \omega_{b,l}\langle m_3^i m_3^{i+1}(1 - n_1^i - m_1^i)(1 - n_2^i - m_2^i)\rangle + \omega_{b,l}\langle m_1^i m_1^{i+1}(1 - n_3^i - m_3^i) + m_2^i m_2^{i+1}(1 - n_3^i - m_3^i)\rangle$

At the loading site (i = 0), i.e., left boundary at track t, Kinesins 'a' and 'b' enter with influx (entry rate) of α_a *and* α_b respectively.

7. $\frac{d\langle n_t^0\rangle}{dt} = \alpha_a\langle 1 - n_t^0 - m_t^0\rangle - \langle n_t^0(1 - n_t^1 - m_t^1)\rangle - \omega_{a,l}\langle n_1^0 n_1^1(1 - n_{t-1}^0 - m_{t-1}^0)(1 - n_{t+1}^0 - m_{t+1}^0)\rangle + \omega_{a,l}\langle n_{t-1}^0 n_{t-1}^1(1 - n_t^0 - m_t^0) + n_{t+1}^0 n_{t+1}^1(1 - n_t^0 - m_t^0)\rangle$

8. $\frac{d\langle m_t^0\rangle}{dt} = \alpha_b\langle 1 - n_t^0 - m_t^0\rangle - \langle m_t^0(1 - n_t^1 - m_t^1)\rangle - \omega_{b,l}\langle m_1^0 m_1^1(1 - n_{t-1}^0 - m_{t-1}^0)(1 - n_{t+1}^0 - m_{t+1}^0)\rangle + \omega_{b,l}\langle m_{t-1}^0 m_{t-1}^1(1 - n_t^0 - m_t^0) + m_{t+1}^0 m_{t+1}^1(1 - n_t^0 - m_t^0)\rangle$

While at the last site (i = N) i.e., right boundary at track t, Kinesins 'a' and 'b' gets delivered at outflow rate of β_a and β_b respectively.

9. $\frac{d\langle n_t^N\rangle}{dt} = \langle n_t^{N-1}(1 - n_t^N - m_t^N)\rangle - \beta_a\langle n_t^N\rangle$

10. $\frac{d\langle m_t^N\rangle}{dt} = \langle v m_t^{N-1}(1 - n_t^N - m_t^N)\rangle - \beta_b\langle m_t^N\rangle$

The equations form a building block to the analytical or numerical solution methodology to our heuristic. For multiple motors, the correlation between the two motors increases the complexity of the equation to O(N^2). Due to influence of external parametric conditions like processivity and lifetime of motors in PDEs, we have not given any analytical or numerical solution and focused on an algorithmic approach as discussed in the Materials and Methods.

2 Supplementary Figures and Tables

Supplementary Figure 1: **Temporal motor output from injury linked 8 µm MMLS scenarios**

Different scenarios and their restrictions dictate the final temporal output of kinesins along the axon length. The figure shows the temporal outflow of motors from different lanes of a combined multi-MMLS. Maximum MT lengths of 8 µm. As in Figure 6, in (**A, B**) the left figure illustrates motor delivery either as "DETAILED" per 40ms time step or "BINNED" within a one second timeframe, equivalent to 25 iterations. **(A – right)** The rotated scatter plots show a varied range of dynamicity of motors for different Scenarios. In multi-MMLS Scenario 1-4P-7D, we see that the outflow of motors is equally distributed along the three parallel MT lanes of Section 1 (Scenario 1). Compared to 4 µm MT maximal length, the temporal outflow rate is reduced to ~ 2 – 3 motors/sec. The overall patterns for output of motors remain relatively unchanged for the remaining segments in both 1-4P-7D and 1-7D-4P, compared to 4 µm maximal lengths in Figure 6.

Supplementary Figure 2: **Impact of Local ATP Concentration on temporal motor output from injury linked 4 µm MMLS scenarios**

Disrupted Axonal transport impacts local mitochondrial distribution and thus regional ATP levels. Here we evaluate different scenarios and their restrictions in the context of regions of low (injury-zone) and high (normal-zone) ATP levels. Maximum MT lengths of 4 µm. As in Figure 6, in (**A, B**) the left figure illustrates motor delivery either as "DETAILED" per 40ms time step or "BINNED" within a one second timeframe, equivalent to 25 iterations. **(A – right)** The rotated scatter plots show a varied range of dynamicity of motors for different Scenarios. The simulation output reveals that when availability of ATP is reduced there is a delayed output flow of both motor types, with higher impact on Kin 1(S) low processivity kinesin. Local ATP Concentration alters the output flow pattern with a larger damaged scenario (1-7D-4P) for regions 7D and downstream 4P.

Supplementary Video 1: Kin1 (S) and Kin3 (F) distribution along proximal staggered MT MMLS track and productive reservoir.

Kinesin dynamics in a staggered constrained MMLS layout for Scenario 4, which has 25% proximal staggered adjacent lanes. Both motors are capable of lateral movement within and along the three MTs. The Kin3 (F) (‘Blue’ marker) kinesins move without hindrance at the initial setup phase but with the onset of Kin1 (S) (‘Red’ marker), they are hindered in their progression and rapid on-off dynamics exchange with the productive reservoir occurs (lower panel ‘Blue peaks’). Kin (S) motor progression is primarily limited by their shorter processivity which is observed as intermediate ‘Red peaks’. Upon availability of adjacent lanes, on close observation, we can observe lateral jump of motors along all three MT lanes 1 ⇋ 2 ⇋ 3 ⇋ 1. The video shows an initial setup phase of system (~5-10 secs) followed by a constant flow dynamic through the end of the simulation.

Supplementary Video 2: Kin1 (S) and Kin3 (F) distribution along distal staggered MT MMLS track and productive reservoir.

Kinesin dynamics in a staggered constrained MMLS layout, for Scenario 7, which has 25% distal staggered adjacent lanes. Both motors are capable of lateral movement within and along the three MTs. The kinesins from start of the simulation are equally distributed along the three MT tracks. The Kin3 (F) ('Blue' markers) motors move freely avoiding collision with Kin1 (S) ('Red' markers), by utilizing lateral jumping to adjacent tracks (1⇋2 ⇋3 ⇋1). Upon distal staggering Kinesins are forced to channelize into a singular lane, which hinders progression of faster motors crowded together with slower motors (spike of ‘Blue Peaks’ beyond staggering point). The congregation of motors onto a singular track results in dynamic on-off activity of both Kin1 (S) and Kin3 (F) to and from the productive reservoir, hindering overall flow rate. The video shows an initial setup phase of the system (~5-10 secs) followed by a constant flow dynamic until the end of the simulation.

Supplementary Video 3: Temporal motor output flow of Kin1 (S) and Kin3 (F) for a proximally staggered MMLS.

Scenario 4 is modeled, which has a 25% proximal staggered MT track but with provision of lateral MT access. In the video the time axis is plotted horizontally, and the vertical axis represents the temporal output of the kinesins from different tracks. Each track is shown separately on the graph, and the output of the kinesins is plotted over time for each track. At the initial setup time (~10 secs) we observe no output, but with gradual flow of time, we see the outflow dominated by Kin3 (F) (‘Blue’) as expected from its motility and processivity. But over time, as Kin1 (S) progress along the track, the flow of Kin3 (F) is suppressed/delayed (~10-15 secs) due to initial crowding by the presence of the slower Kin1 (S) motors. The outflow distribution reflects Kin3 (F) prominence on less crowded lanes 1 and 3. The majority of Kin1 (S) is concentrated in the central lane due to their unhindered progression. We observe this MT distribution becomes more equally distributed over time, denoting a constant equilibrium flow dynamic of motors along MT tracks.

Supplementary Video 4: Temporal motor output flow of Kin1 (S) and Kin3 (F) for a distal staggered MMLS.

Scenario 7 is modeled, which has a 25% distal staggered MT track with the provision of lateral MT access. In the video the time axis is plotted horizontally, and the vertical axis represents the temporal output of the kinesins from different tracks. Each track is shown separately on the graph, and the output of the kinesins is plotted over time for each track. At the initial setup time (~10 secs) we observe no output, but with gradual flow of time, we see the outflow dominated by Kin3 (F) ('Blue') that dominates the outflow. As Kin1 (S) progresses along the track, the flow of Kin3 (F) is slightly suppressed/delayed (~10-15 secs) due to initial crowding by the slower Kin1 (S). Upon channelization to a single track the

output is also restricted to the middle track for both Kin1 (S) and Kin3 (F). The observed behavior demonstrates the significance of distal channelization in regulating the output flow of kinesins and cargos from axonal MT tracks.
