# Supplementary figures and images for: Axonal transport during injury on a theoretical axon
Source: Front Cell Neurosci. 2023 Aug 11;17:1215945. doi: 10.3389/fncel.2023.1215945 (PMC10450981; doi:10.3389/fncel.2023.1215945)

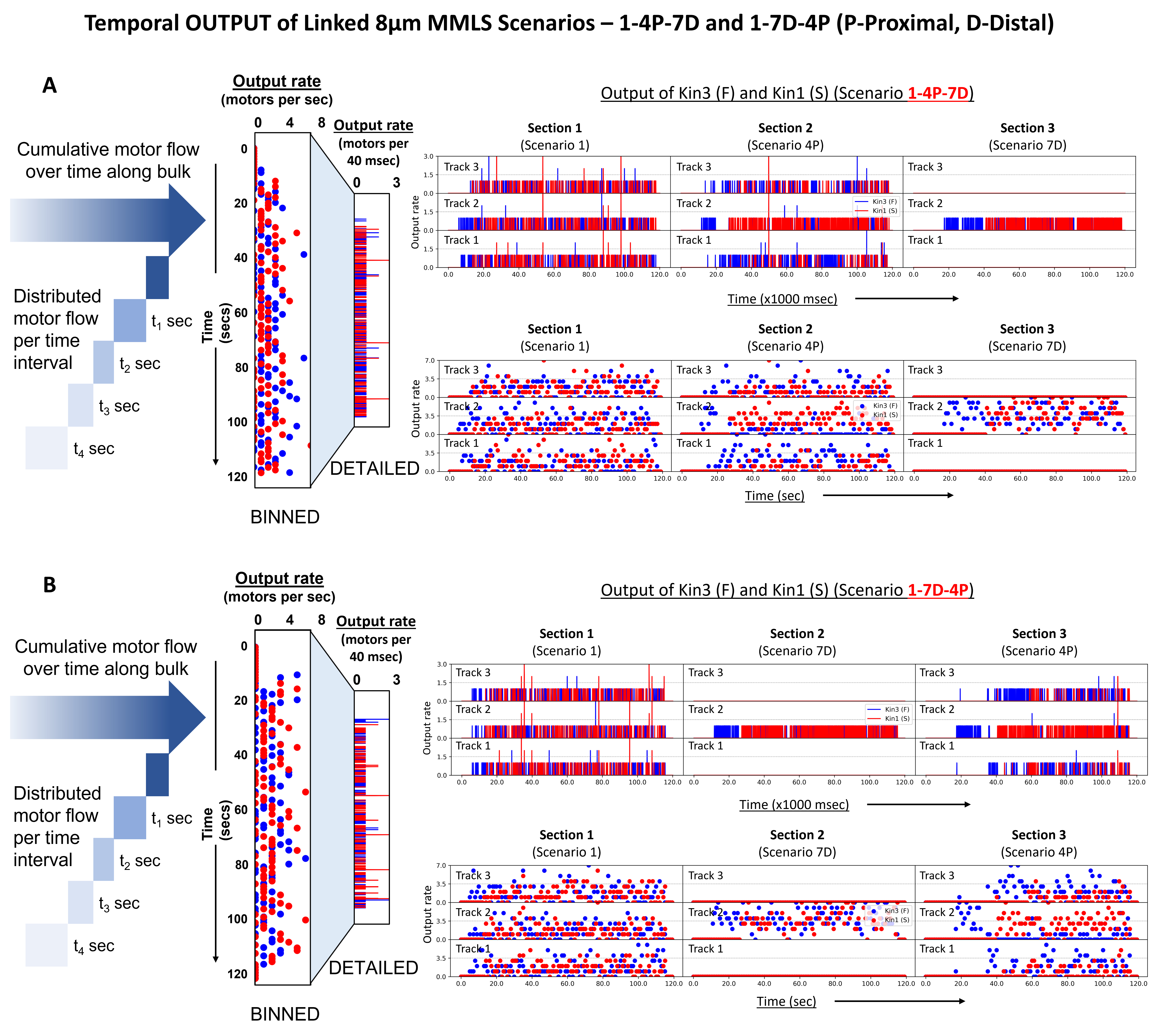

Supplement: Supplementary file 6 [file Image_1.JPEG]

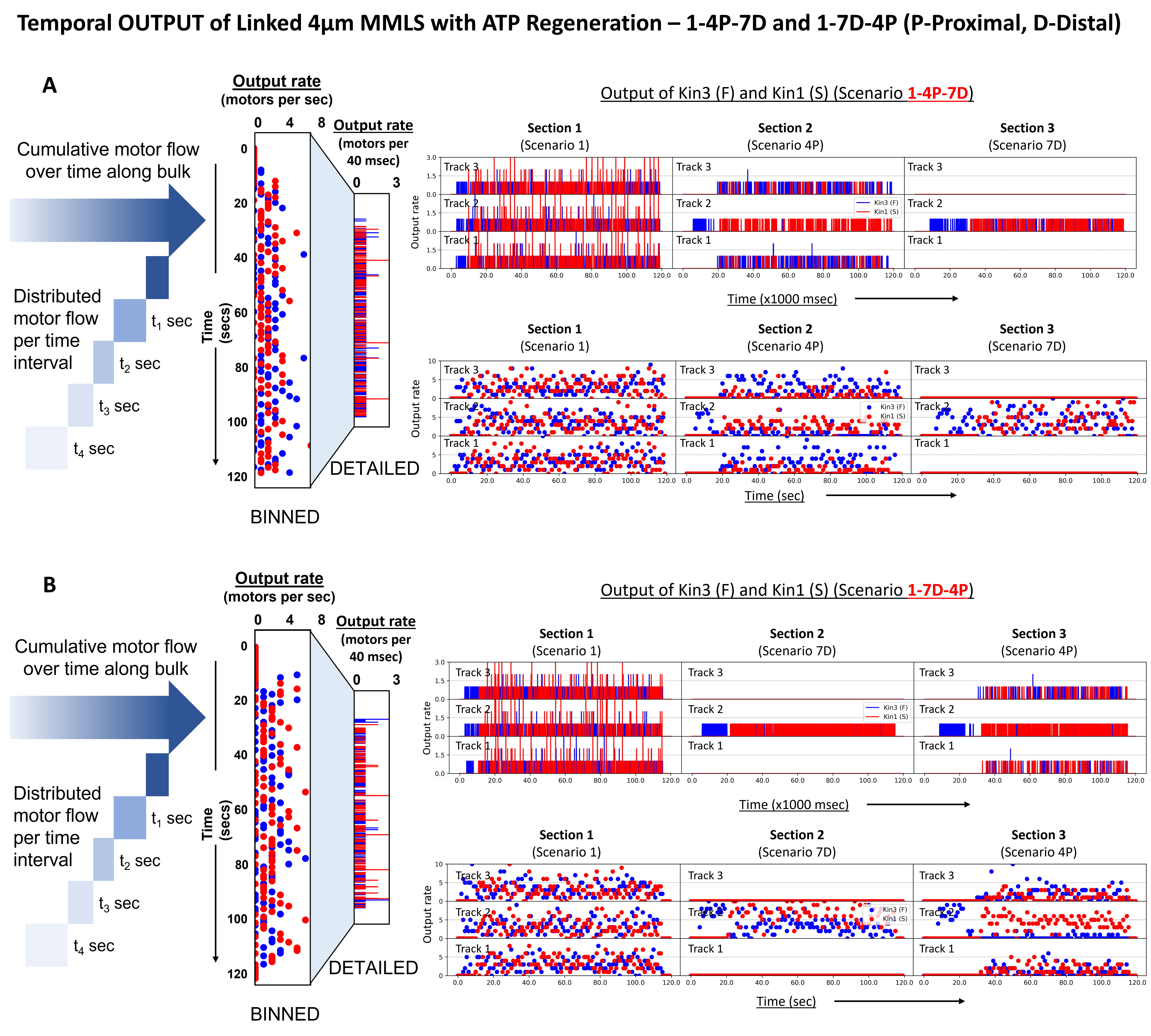

Supplement: Supplementary file 7 [file Image_2.JPEG]
